# Whole-exome sequencing identifies a missense variant in the cytoskeletal gene *SVIL* implicating actin remodeling in COVID-19 severity in a Pakistani cohort

**DOI:** 10.1371/journal.pone.0360429

**Published:** 2026-10-12

**Authors:** Madeeha Sadiq, Bushra Wasim, Ambrina Khatoon, Ashok Kumar, Nida Hussain, Urfa Tabassum Kayani, Talat Mirza

**Affiliations:** 1 Department of Anatomy, Ziauddin University, Karachi, Pakistan; 2 College of Molecular Medicine & Research Department, Faculty of Health Sciences, Ziauddin University, Karachi, Pakistan; 3 Department of Pulmonology, Ziauddin Hospital, Karachi, Pakistan; 4 Department of Medicine, Ziauddin Hospital, Karachi, Pakistan; 5 Department of Research, Faculty of Health Sciences, Ziauddin University, Karachi, Pakistan; Università degli Studi di Parma: Universita degli Studi di Parma, ITALY

## Abstract

**Background:**

The current study investigated host genetic associations with disease severity in COVID-19 patients of South Asian origin and explored its molecular basis.

**Methods:**

Covid-19 patients were stratified into disease severity groups based on the clinical and radiographic findings. Genomic DNA was extracted from peripheral blood samples. A two-step discovery and validation approach was utilized for genetic analysis. Whole-exome sequencing (WES) based variant prioritization was performed in a discovery cohort of severe disease patients followed by validation in an independent cohort. Bioinformatics analysis including data alignment, and annotation to the GRCh38 human reference genome was done by *Ensembl VEP* software. WES filtration pipeline utilizing predicted pathogenicity and population allele frequencies focused on the low-frequency and rare variants to filter out the variants of concern. Computational predictive tools and simulations were utilized for the structural and functional analysis of the identified high-risk SNP. Statistical analyses were done by SPSS version 24 and GraphPad Prism version 10.

**Results:**

The missense variant rs199726033 of *SVIL* gene demonstrated the highest recurrence in the WES discovery cohort. The minor allele frequency (29.2%) was substantially higher than that reported for South Asians in the *gnomAD* v4 database (1.051%). A significant association of the mutant AC genotype of rs199726033 and disease severity was found in the validation cohort. Protein modeling and structural analysis localized the variant within a functionally conserved Gelsolin domain. Moreover, functional and pathway analysis of the *SVIL* variant indicates its potential role in actin filament remodeling relevant to COVID-19 related vasculopathy and cytoskeleton dynamics thus influencing host-viral interactions.

**Conclusion:**

A *SVIL* missense variant is associated with disease severity and may influence host cytoskeletal processes relevant to COVID-19 severity.

## 1. Introduction

The COVID-19 pandemic has provided a rare opportunity to scientists across the globe to understand better the disease related host genetic variability among populations. The global disparity in disease outcomes, severity and mortality rates were quite distinct throughout the course of the pandemic. While these differences can be partially ascribed to different factors such as variations in government responses, healthcare infrastructures, population densities, and varying age compositions of different populations, the genetic architecture of populations likely contributed significantly [1,2]. Substantial inter-country variation in mortality was documented in the South-Asian region, with India reporting the third-highest death toll by early 2024 [3]. This contrasts sharply with reports of relatively low mortality in Pakistan despite both countries sharing close genetic similarities [4]. Although the pandemic status of COVID-19 was lifted in mid-2023, the WHO and global scientific community emphasize that its transition to endemicity does not rule out chances of future disruptive infectious waves, similar to patterns seen in other endemic diseases like malaria and measles [5]. Therefore, continued research aiming to further explore the molecular and genetic basis of this viral disease can prove helpful in deciphering the disease pathogenesis deeply. COVID-19 disease trajectory comprises the clinical spectrum from completely asymptomatic cases to critically ill patients requiring ICU care and ventilatory support [6]. Extensive research during the pandemic has been focused on elucidating the pathophysiology and different factors affecting this disease severity. Both non-genetic and genetic host attributes are associated with disease severity and clinical course. Notably, well-established non-genetic risk factors include male sex, advanced age, and underlying comorbidities [7,8]. However, over the course of the pandemic, it was noted that disease severity was not confined to patients belonging to these demographic groups alone [7]. Thus, making it increasingly evident that host genetic factors play a key role at different steps of infection and disease progression [8]. Research has highlighted the crucial role of a myriad of genes in COVID-19 pathophysiology. Various genes, including *ACE2, TMPRSS2, DPP9, LZTFL1, TYK2, IFNAR2, CCR2,* and others, appear to be crucially involved at different steps of viral cell entry, viral replication, innate immune efficiency, cytokine storm manifestation, and in mounting the resultant host immune response [8–11]. Researchers from across the globe have identified genetic variation at multiple genomic sites to be involved in the clinical picture of respiratory diseases. The genetic landscape of an individual, particularly and that of a population generally, appears to play a key role in generating the COVID-19 dynamics [8]. The existence of many single nucleotide polymorphisms (SNPs) in the population may increase infection susceptibility or cause an excessive inflammatory response [11–13].

Recent studies exploring the genetic basis of COVID-19 have highlighted the impact of common genetic variants with minor allele frequency, MAF ≥ 5% as well as low-frequency, rare, and ultra-rare genetic variants with MAF < 5% on disease severity [14]. Several studies have reported an association of rare and ultra-rare genetic variants with disease phenotypes [15–17]. These observations support the omnigenic model of complex traits. This model postulates that there are regulatory interactions across the genome which span across the core genes involved in any disease pathogenesis and also certain genes that are relevant to some peripheral pathways, both these types of genes act as contributors to disease mechanisms [18,19]. Studies report enrichment of rare, high-impact missense variants in severe COVID-19 cases, particularly in primary immunodeficiency genes and their functional partners [18,20–22]. Integrative models have been proposed combining common driver polymorphisms and rare, high-penetrance variants to predict clinical severity [19,23]. A recent study incorporated both common and rare genetic variants including sex-specific loci to develop an integrated polygenic score which showed promising predictive potential for disease severity [24]. Thus, recent research in this context substantiates the omnigenic concept and suggests that research emphasis should not be restricted to a few common genes only, but a complex interplay of diverse, less frequent, more penetrant genetic variants should also be explored and probed into especially in the context of differing population genetics. Thus, digging deeper into the genetic architecture and the related molecular mechanisms may potentially guide diagnosis and treatment strategies by indicating that the genetic foundation of COVID-19 severity involves a complex interaction of several kinds of genetic variants throughout the genome.

The COVID pandemic bolstered research into viral pathogenesis, host-pathogen dynamics and the translational effects of these interactions on clinicopathological disease mechanisms. Several studies have explored the viral genome and viral variants affecting the Indian subcontinent [25,26]. But there is a dearth of data on host genetics from this part of the world in the context of COVID-19. Research exploring the unique genetic landscape of the Pakistani population can offer valuable insights into disease mechanisms and viral-host interactions that shaped a distinct pandemic trajectory in the Indian subcontinent, differing from patterns observed in other regions of the world [27].

The genetic makeup of the Pakistani population in the context of Covid 19 disease phenotype has been explored by a few studies, but most of them have focused on variants reported for European and Caucasian populations. Multiple studies published from the Indian subcontinent have specifically targeted *ACE2* and *TMPRSS2* genes, which are probably the most widely studied in the context of COVID-19 phenotypes [28,29]. The frequency of *ACE2* and *TMPRSS2* gene variants was found to be comparatively lower in the Asian population as compared to Western populations [30]. Also, fewer variants were significantly associated with disease severity [28,29,31]. These findings point towards the need to analyze the role of low-frequency and rare variants in the population-specific context, an area particularly unexplored in Pakistani patients. Accordingly, this study is focused to understand the molecular and genetic mechanisms of host-pathogen interactions in the backdrop of the associated clinical implications.

## 2. Materials and methods

### 2.1. Study subjects

The study design was cross-sectional and research approval was granted by the institutional review board of Ziauddin University (Reference code: 0010421AKRES). The study was conducted in accordance with the Declaration of Helsinki. Sample size was calculated using OpenEpi calculator (version 3.01). Based on reported prevalence of 8.3% in Pakistan [32]. With a 95% confidence level and 5.5% absolute precision, the calculated sample size was 97. This was rounded up to 100 cases. Study subjects were enrolled after obtaining written informed consent by non-probability purposive sampling technique. A total of 108 confirmed COVID-19 out-patient and in-patient samples were collected from three campuses of Ziauddin Hospital between January and August 2022. All participants were SARS-CoV-2 vaccinated. Exclusion criteria included patients having malignant conditions or autoimmune diseases or those who had received chemotherapy and radiotherapy

Standard procedure comprising aseptic protocol was utilized for obtaining a 5 mL blood sample for DNA extraction. After the COVID-19 diagnosis confirmation through RT PCR, relevant clinical data was obtained from the hospital’s electronic medical records. Additional imaging assessments, including Chest X-ray (CXR) and High-Resolution Computed Tomography (HRCT), were performed where required.

CDC guidelines [10] were utilized for patient stratification into disease severity groups based on the clinical signs, symptoms, and radiological findings. Patients were grouped into two disease categories for statistical comparisons, ‘severe’ group included severe and critical cases of COVID-19 infection, while the ‘non-severe’ group included all patients falling in the asymptomatic, mild, and moderate disease groups.

Six patient samples did not meet the required post DNA extraction integrity check and were excluded from further workup bringing the study sample to a total of 102 patient samples. All experiments were performed as per the relevant lab protocols and standard guidelines.

### 2.2. Genomic DNA extraction

Genomic DNA was extracted from EDTA whole blood using the CTAB method. DNA concentration and purity were assessed using a Multisky spectrophotometer and PicoGreen assay, while integrity was evaluated by agarose gel electrophoresis and DNA Integrity Number (DIN) measurement on a 2100 Bioanalyzer (Agilent).

### 2.3. Whole-exome sequencing (WES)

WES was performed for 12 discovery cohort samples using the Illumina platform. 1ug of input gDNA was utilized to generate standard exome capture libraries by the Agilent Sure SelectXT Low Input Target Enrichment protocol. WES libraries were prepared after shearing to a target peak size of 150–200 bp, followed by end-repair, addition of an ‘A’ tail, and adapter ligation to fragments was done and adapter-ligated products were PCR amplified. Further, the libraries were qualified and mixed with hybridization buffers, blocking mixes, RNase block, SureSelect all-exon capture library, according to the standard Agilent SureSelect Target Enrichment protocol (SureSelect V6-Post). The final product was sequenced on NovaSeq6000 system (Illumina) according to the manufacturer’s protocol. Indexed, captured libraries from all the samples were sequenced on the Illumina platform, to generate 150 bp sequence reads at a desired sequencing depth of 100 × . Sequencing data was generated in the form of Fastq files.

### 2.4. Quality control of sequencing data, alignment and variant calling

Sequencing data quality was checked through ‘Fast QC’ (bases with Phred quality scores above 30 were considered). In case of lower quality scores, reads were trimmed and aligned to the GRCh38 using ‘BWA’ (BWA-MEM). PCR duplicates were removed in the resultant BAM file by using Picard MarkDuplicates. Realignment was done by using GATK. GATK Base-Recalibrator was used for recalibration of BAM files. Variant Genotyping was done for the resultant BAM file by Haplotype Caller of GATK, and variant filtration was done by Variant Filtration of GATK Tool for generation of VCF files. To minimize false positive results in the resultant variant data, a minimum quality score threshold of 30 (QUAL < 30) and a minimum depth threshold of 15 (DP < 15) was applied. The VCF files were annotated using the *Ensembl Variant Effect Predictor (VEP)* software [33]. Visualization of raw read alignments was performed using the *Integrative Genomic Viewer (IGV)* to confirm the quality of variant calls [34].

### 2.5. Variant prioritization and pathogenicity prediction

Identification of variants with potential functional consequences was done according to the ACMG (American College of Medical Genetics and Genomics) guidelines [35]. Consequently, variants were filtered according to the pathogenicity predictors and minor allele frequency (MAF). Fig 1 summarizes the variant filtration workflow. To identify variants exhibiting higher pathogenicity prediction three *in silico* prediction tools, CADD (combined annotation dependent depletion), SIFT (sorting intolerant from tolerant), and Polyphen2 (Polymorphism Phenotyping v2) scores (CADD ≥ 15; SIFT: deleterious or tolerated; PolyPhen2: probably or possibly damaging) were utilized.

**Fig 1:**
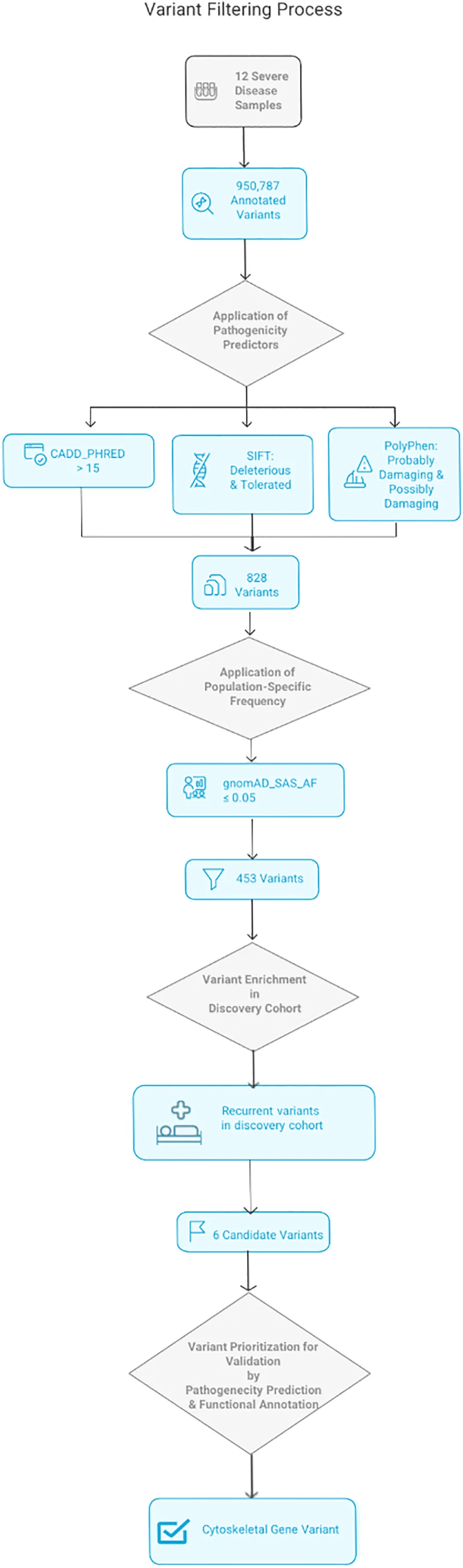
Variant Prioritization Workflow. DNA extracted from the discovery cohort of severe and critical disease patients was subjected to whole-exome sequencing (WES). After quality control, 950,787 annotated variants were obtained. Functionally relevant variants were filtered using pathogenicity predictors (CADD ≥ 15; SIFT: deleterious or tolerated; PolyPhen2: probably or possibly damaging), yielding 828 variants. Population-based filtering using *gnomAD* South Asian MAF ≤ 0.05 retained 453 low-frequency variants. Genotype-based filtration within discovery cohort samples identified six enriched missense variants. Final prioritization was based on functional annotation and pathogenicity prediction, highlighting a cytoskeletal gene variant for further validation.

Population-based filtration was performed using *gnomAD* v4 MAF scores ≤ 0.05 for the South Asian population [36] to retain low-frequency and rare variants. Finally, a genotype-based recurrence filter was applied requiring the alternate allele to be present in at least three of twelve discovery cohort patients.

To identify the variants with elevated frequencies among severe cases and to evaluate variant enrichment within the discovery dataset of severe disease patients, a genotype-based recurrence filter was applied. Variants with alternate alleles present in at least three out of the twelve severe disease cohort patients were prioritized.

### 2.6. Integrative in silico and structural variant analysis

Sequence-level variation was assessed by performing alignments using *Jalview*, followed by visualization and conservation analysis to assess amino acid variability across the selected region. For nucleotide-level evaluation of validation cohort sequencing data, the *Cbioportal Mutation Mapper* was used to annotate the mutation sites [37,38].

Protein-level changes were modeled by generating the SVIL protein three-dimensional PDB structure using the EMBL-EBI ProtVar database [39], an online resource to investigate SNV missense variation in humans. *ChimeraX* was then employed for structural comparison of wild-type and mutant models, including surface pocket analysis to identify conformational shifts caused by the variant [40].

Functional and structural consequences of the missense variant were assessed using multiple computational prediction tools. *Alpha Missense*, a resource hub from the *EMBL* database, was utilized for predicting variant pathogenicity [41]. *Interpro* and *Uniport* databases were explored for annotation of the identified missense variation within the context of relevant protein domains [42,43]. Additionally, the *EMBL-EBI Varsite and MutPred 2* web servers were used for molecular dynamics simulations to assess the variant’s effect on structural features such as coil-coil regions and disordered interfaces [44,45].

Finally, integrative network analysis was performed to explore the broader interaction context using *GeneMANIA* for gene-gene functional interaction network [46] and *Intact* database for the protein-protein interaction mapping [47].

### 2.7. PCR amplification and Sanger sequencing

Polymerase chain reaction (PCR) DNA amplification was done for Sanger sequencing. Primer Blast software was used to design a primer flanking a 420-bp amplicon covering the targeted variant area on Exon 22 of the *SVIL* gene (Chromosome 10, NC_000010.11). Forward primer, 5’GTCTAGGTGCGTGCACATGTAT 3’ and reverse primer 5’ ACTTTGTGGAGTCCAAGACCC 3’ were used. PCR cycles were run with initial denaturation at 95°C for 5 min, followed by 35 cycles of 1 min at 95°C, 1 min at 62.5°C, and 1 min at 72°C with a final extension of 5 min at 72°C. Submerged 2% agarose gel electrophoresis was used to observe the amplification products. After purification of amplification products, Sanger sequencing of the 420 bp amplicons was performed.

### 2.8. Statistical analysis

For statistical analysis, SPSS version 24 and GraphPad Prism version 10 were used. Qualitative data were expressed as frequencies and percentages. Quantitative variables were presented by descriptive statistics, including mean, standard deviation (SD), median, and interquartile range (IQR). Normality of quantitative variables were assessed by the Shapiro-Wilk test. For normally distributed data, independent t-tests were used to compare means between groups. For non-normally distributed data, the Mann-Whitney U test was used. Fisher’s Exact/ Chi square test was applied for initial comparison between allele frequencies of severe and non-severe groups. Variants showing statistical significance in this initial screening were further evaluated using univariate logistic regression. Variants with p-value ≤ 0.2 in the univariate model were subsequently entered into a multivariate logistic regression model, adjusted for age, gender, and comorbidity status, to identify independent predictors of severe disease, p-value ≤ 0.05 was considered statistically significant.

## 3. Results

### 3.1. Patient statistics

The study patients’ clinical and demographic characteristics are summarized in Table 1 (Individual level data is provided in S1 Table). A total of 102 COVID-19 patients were included in the study after DNA integrity check. Among these 58(56.86%) had severe disease, and 44(43.13%) were characterized as non-severe patients. Severe patients were significantly older (median 73 years contrary to 56 years, p < 0.001), had higher respiratory rate (p < 0.001), lower oxygen saturation (p < 0.001) and significantly lower hemoglobin levels as compared to the non-severe patients (p = 0.041). Among serum electrolytes, chloride and urea levels were significantly elevated in severe patients (p = 0.018 and p = 0.003, respectively). Regarding comorbidities, hypertension and diabetes were significantly more prevalent in the severe group (p = 0.009 and p = 0.002, respectively). Clinical outcome was significantly worse in severe patients, with 24.0% mortality compared to 2.3% in the non-severe group (p = 0.005). No significant differences were observed between groups for pulse, platelet count, leukocyte count, sodium, potassium, bicarbonate, CRP, or IHD prevalence.

**Table 1:** Clinical and demographic features of COVID-19 patients.

*Parameter*	*Total participants (N = 102)*	*Severe patients (N = 58)*	*Non-Severe* *patients (N=44)*	*P-value*
Age (years)†	68.0 (56.0-80.25)	73.0 (62.8-84.0)	56.0 (3.5-73.5)	<0.001*
Gender (Female/Male)	52 (51) / 50 (49)	32 (55.2) / 26 (44.8)	20 (45.5) / 24 (54.5)	0.319
Hb (g/dl) ‡	11.2 ± 2.1	10.9 ± 2.0	11.7 ± 2.2	0.041*
Leukocyte count (× 10^9^/L) ‡	10.9 ± 4.9	10.6 ± 4.9	10.92 ± 4.7	0.809
Platelet count (× 10^9^/L) ‡	204.9 ± 76.4	202.9 ± 73.8	206.3 ± 83.8	0.714
O_2_ Saturation (%) †	89.0 (75.0-90.0)	75.0 (67.5-87.0)	97.5 (95.0-98.0)	<0.001*
Pulse (beats/min) †	88 (75-102)	88 (80-102)	81 (81–89)	0.144
Resp rate (breaths/min) †	22 (20–30)	26(22–30)	21 (20–22)	<0.001*
***Serum Electrolytes***				
Sodium (meq/l) ‡	138.9 ± 4.9	138.6 ± 5.6	137.36 ± 3.6	0.98
Potassium (meq/l) †	4.0 (4.0-4.6)	4.1 (4.0-4.8)	4.0 (3.8-4.6)	0.301
Chloride (meq/l) †	102.0 (97.0-105.8)	103.0 (97.6-108)	101.0 (95–104)	0.018*
Bicarbonate (meq/l) †	23.0 (19.5-25.3)	23.0 (18.7-25.3)	22.6 (20.0-25.8)	0.755
Urea (mg/dl) †	67.5 (41.0-109.0)	82 (44-118.3)	44.5 (25.8-83.0)	0.003*
Creatinine (mg/dl) †	1.3 (0.9-1.9)	1.6 (0.9-2.3)	1.1 (0.8-1.5)	0.053
CRP (mg/L) †	63.8 (33.0-177.7)	74.1 (36.2-179.2)	46.25 (11.7-138.7)	0.128
D-Dimer (ng/ml) †	1105(328-1881)	1316(734-2598)	442(56-758)	<0.01*
***Comorbidities*** present/absent	83(81.4) / 19 (18.6)	55 (94.8) / 3 (5.2)	28 (63.6) / 16 (36.4)	<0.001*
Hypertension present/absent	59 (57.8) / 43 (42.2)	40 (69.0) / 18(31.0)	19 (43.2)/ 25 (56.8)	0.009*
Diabetes present/absent	57 (55.9) / 45 (44.1)	40 (69.0) / 18 (31.0)	17 (38.6) / 27 (61.4)	0.002*
IHD present/absent	36(35.3)/ 66(64.7)	25(43.9)/32(56.1)	11 (24.4)/34(75.6)	0.077
**Clinical outcome** Death/Recovered	15(14.7)/87(85.3)	14(24.0) / 44 (76.0)	1 (2.3) / 43 (97.8)	0.005*

Normally distributed numeric variables are marked with ‡ and presented as Mean ± SD (Independent Sample T-test). Not normally distributed numeric variables are indicated by † and presented by Median (IQR) (Mann-Whitney U Test). Categorical variables are presented as N (%) and were compared using Chi-square or Fisher’s exact test. Statistically significant results (p < 0.05) are highlighted in bold and marked with an asterisk (*).

CRP: C-reactive Protein, IHD: Ischemic heart diseases

### 3.2. WES results

Sequencing quality showed that approximately 94% was above Q30 (Phred quality score > 30). Mean depth of target regions was approximately 65-67x. The target regions exhibited high coverage, with ~ 99% having at least 1x coverage and ~ 90% having at least 20x coverage. A total number of 950,787 annotated variants were observed and analyzed in 12 patient samples (Fig 1, S2 Table). The chromosomal distribution of variants is displayed in S1 Fig. Among coding consequences synonymous and missense variants predominated (Fig 2a). Pathogenicity prediction yielded 828 pathogenic variants. Population-based filtration (MAF scores ≤ 0.05) resulted in the identification of 453 low-frequency and rare variants. Six missense variants were identified after genotype-based recurrence filtering (alternate allele present in ≥ 3 of 12 discovery cohort patients) (Table 2). The missense variant rs199726033 (NC_000010.11:g.29490918A > C) in the *SVIL* gene exhibited the highest frequency in the WES samples. IGV visualization of the raw WES alignments at the variant locus (chr10:29,490,918) confirmed clean, symmetric read coverage across both strands. No evidence of mapping gaps or low quality read end clustering was observed (S2 Fig). This variant was selected for subsequent validation tests in the remaining samples based on its strong biological plausibility as indicated by functional annotation and pathogenicity prediction analyses.

**Table 2:** Functional annotation and frequency of six variants identified in the WES cohort.

Variant ID	Gene	Chromosomal location	Type of variant	Carrier Frequency in WES Samples (n/N)	MAF in the South Asian Population (*gnomAD* v4 database)	CADD	Polyphen	SIFT
**rs199726033**	***SVIL***	**10:29490918-29490918**	**Missense**	**7/12**	**0.01051 (1.051%)**	**26.4**	**PD (0.518)**	**deleterious (0)**
rs62123481	*GGN*	19:38384432-38384432	Missense	4/12	0.02007 (2.007%)	24.1	PD (0.956)	deleterious (0)
rs112658494	*KLK14*	19:51079551-51079551	Missense	3/12	0.01036 (1.036%)	22.8	PD (0.927)	deleterious (0.04)
rs138878772	*NRG3*	10:82985465-82985465	Missense	3/12	0.01077 (1.077%)	27.2	PD (0.992)	deleterious (0)
rs140699573	*CHST6*	16:75478836-75478836	Missense	3/12	0.0207 (2.07%)	24.7	PD (0.996)	deleterious (0)
rs555368347	*CCDC88B*	11:64343799-64343799	Missense	3/12	0.009942 (0.9942%)	–	PsD (0.612)	deleterious (0.02)

* PD = Probably Damaging, PsD = Possibly Damaging, MAF = Minor Allele Frequency, WES = Whole Exome Sequencing, n/N = number of samples with alt allele/ total number of discovery cohort samples

**Fig 2:**
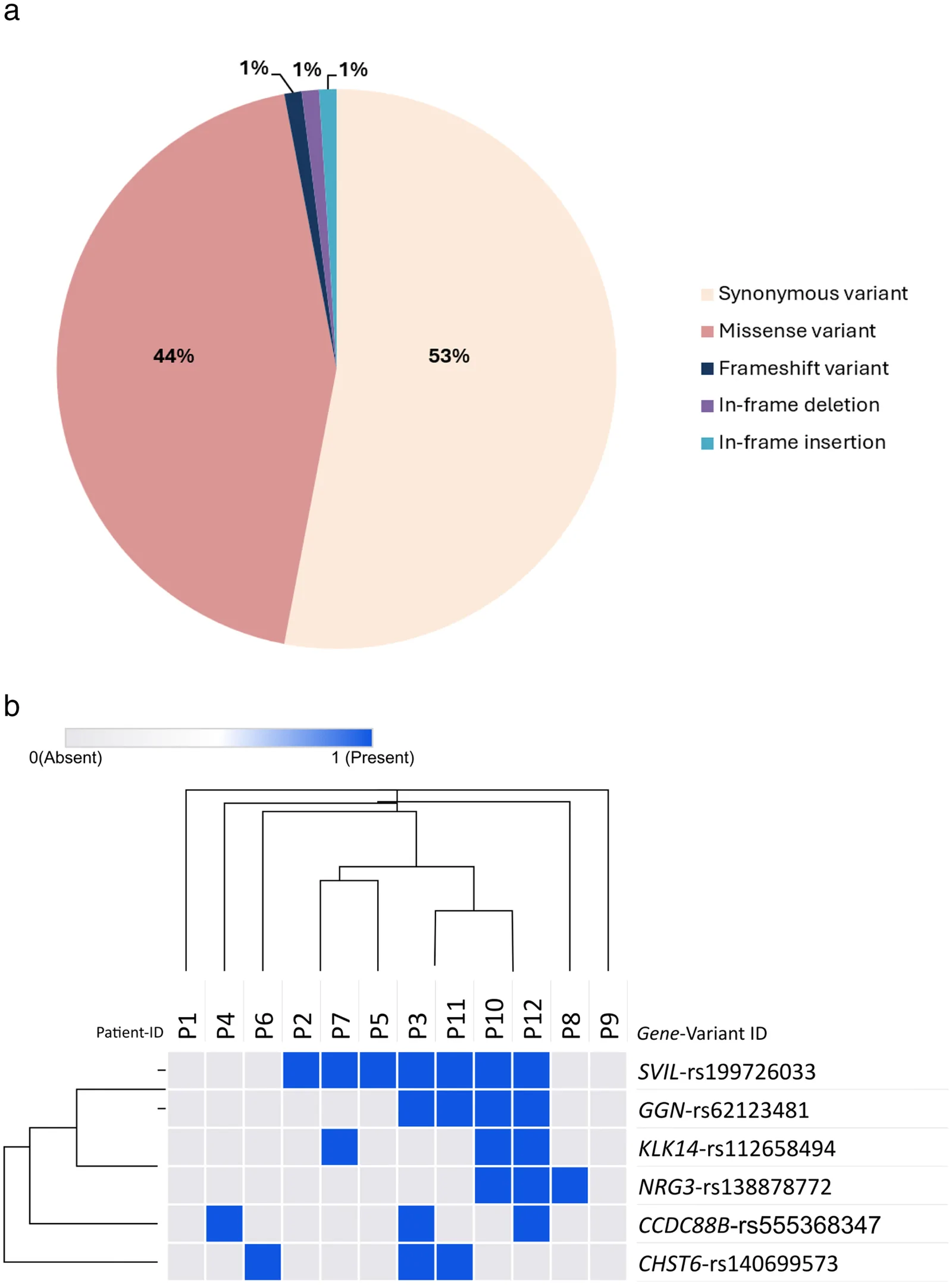
Whole Exome Sequencing Results (a) Distribution of coding consequences in WES discovery cohort, pie chart reflects predominance of synonymous and missense variants (b) Heatmap displaying distribution of six prioritized missense variants across the 12 WES discovery cohort patients. Blue indicates presence while absence of alternate allele is represented by grey color. (Heatmap data is provided in S3 Table) *SVIL*- rs199726033 was the most frequent variant (7/12 patients, 58.3%). A co-occurrence block of *SVIL, GGN, KLK14* and *NRG3* is observed in patients (P3, P11, P10, P12).

### 3.3. Sanger sequencing results

In the validation cohort 90 patients were included in which 46 samples were from the severe(S) group and 44 samples from the non-severe (NS) disease group. Fifty variants were analyzed, including 40 missense, 7 intronic, 2 synonymous and 1 splice site variant (Fig 3a, S4 Table). Twenty-seven variants exhibited MAF > 0, and nine demonstrated statistically significant differences in frequency between the severe and non-severe disease groups based on the initial Fisher’s exact test. Multivariate logistic regression, adjusted for age, gender, and comorbidity status, was performed on the nine variants to validate their association while controlling for major clinical confounders. Only two variants, rs199726033 and rs45519332, remained statistically significant as independent predictors of severe disease in the multivariate model (Table 3 & Fig 3b). Comprehensive Sanger variant details, including genotype counts, allele frequencies, and statistical results, are provided in S4 and S5 Tables.

**Table 3:** Association of SVIL gene variants with severe COVID-19 in the validation cohort.

Variant ID	Genomic Position (GRCh38)	Nucleotide Change (Ref > Alt)	Amino Acid Change	Type of Variant	Unadjusted p-value†	Adjusted Odds Ratio	95% CI	Adjusted p-value
**rs199726033**	**10:29490918**	**A > C**	**Leu1374Arg**	**Missense**	**0.0047**	**4.07**	**1.04 −15.93**	0.044 *
rs1391759737	10:29490990	C > T	Arg1350Gln	Missense	0.0123	2.72	0.27 −26.99	0.393
rs184191419	10:29490933	G > A	Ala1369Val	Missense	0.0073	2.46	0.57 −10.60	0.227
**rs45519332**	**10:29491007**	**G > A**	**Ser1344=**	**Synonymous**	**0.0271**	**6.17**	**1.05 −36.32**	0.044 *
rs540949405	10:29491024	G > C	–	Splice Site	0.0264	–	–	–
rs74130727	10:29490691	C > T	–	Intronic	0.0264	–	–	–
rs746330670	10:29491014	G > A	Thr1342Met	Missense	0.0026	–	–	–
rs750205669	10:29490864	C > T	Arg1392Gln	Missense	0.0026	–	–	–
rs757300194	10:29490860	C > T	Met1393Ile	Missense	0.0026	–	–	–

p-values were obtained using Fisher’s Exact Test (†). Variants with p < 0.05 were further analyzed in univariate logistic regression; those with p < 0.20 were included in multivariate models adjusted for age, sex, and comorbidities. The equals sign (=) denotes a synonymous variant, and a dash (—) indicates that the variant did not meet inclusion criteria for subsequent analysis. Variants that showed significant association with severe COVID-19 in the adjusted analysis (p < 0.05) are indicated in bold.

**Fig 3:**
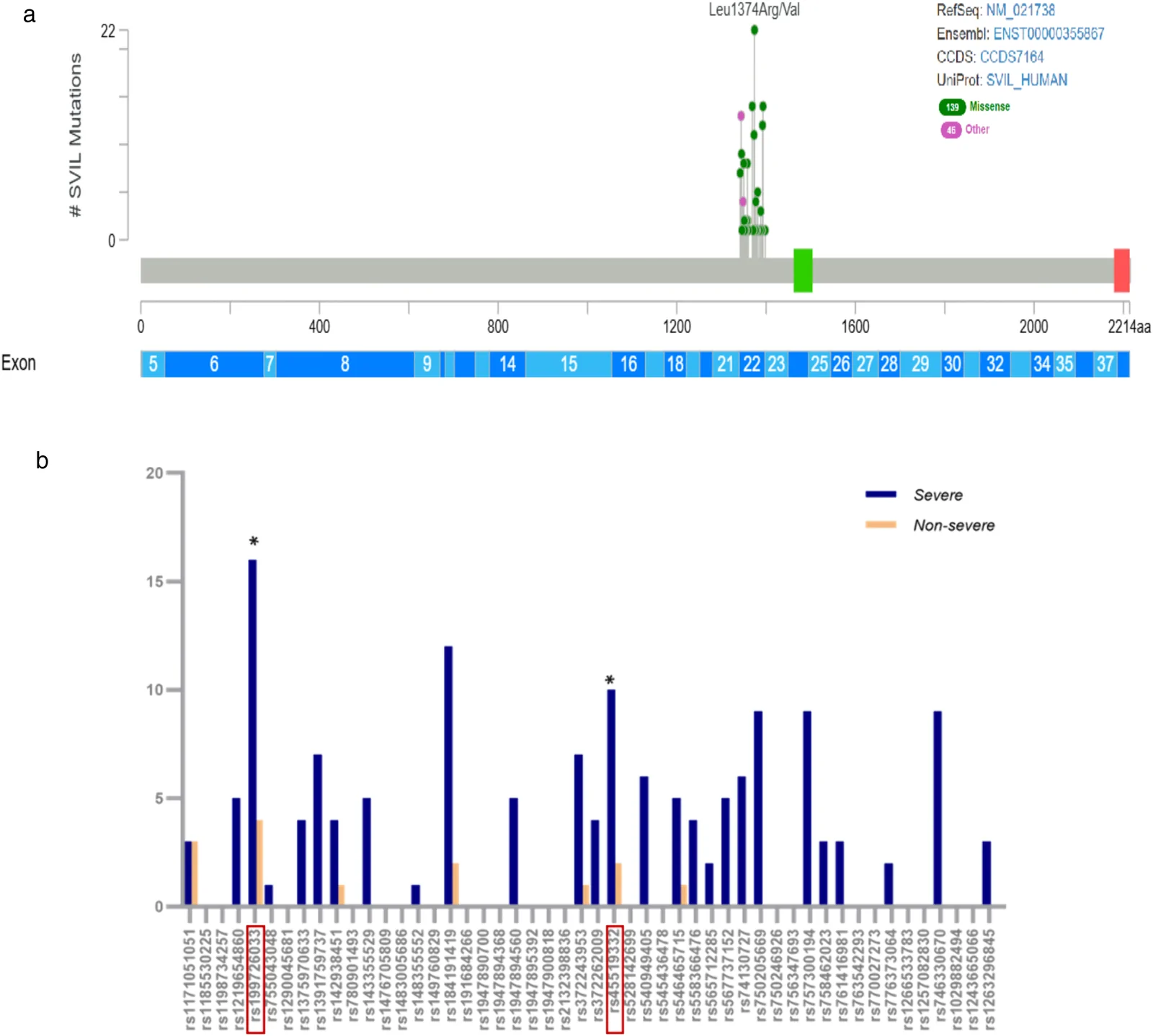
Validation cohort sequencing results (a) Variant distribution across the SVIL gene in the validation cohort. The blue bar marks SVIL exons; the X-axis denotes amino acid positions, and the Y-axis denotes the number of samples carrying each mutation. Green dots indicate missense variants; purple dots represent other types. Position 1374 (Leu1374Arg/Val) was the most frequent mutated site, detected in 20 samples (Fig created by Mutation Mapper on Cbioportal, S4 Table) [38]. (b) Frequency distribution of genetic variants among severe and non-severe COVID-19 cases. Bars show the number of individuals carrying each variant (blue: severe; orange: non-severe). Two SVIL variants, marked with red boxes, were independently associated with disease severity (Multivariate logistic regression applied after adjusting for potential confounders, p-value < 0.05 [*]) S5 Table).

### 3.4. Structural modeling and predicted functional impact of rs199726033

To assess the structural and functional impact of the variant computational protein structural modeling was done. The variant rs199726033 causes a missense substitution of amino acid leucine-to-arginine at amino acid position 1374 (L1374R), potentially altering the SVIL protein structure and function. Jalview was utilized for the initial nucleotide level sequence alignment and subsequent translation into the corresponding amino acid sequences. Owing to the location of the SVIL gene on the reverse strand, the sequences were reverse-complemented before translation to ensure correct coding orientation. The resulting Jalview alignment revealed that the L1374R mutation lies within a relatively conserved stretch of amino acids (S3 Fig). Protein modeling and structural visualization revealed that this residue lies within the P55 binding pocket region of SVIL, which is implicated in protein–protein interactions (Fig 4). This structural context suggests that alterations at this site could influence the protein’s interaction dynamics.

**Fig 4:**
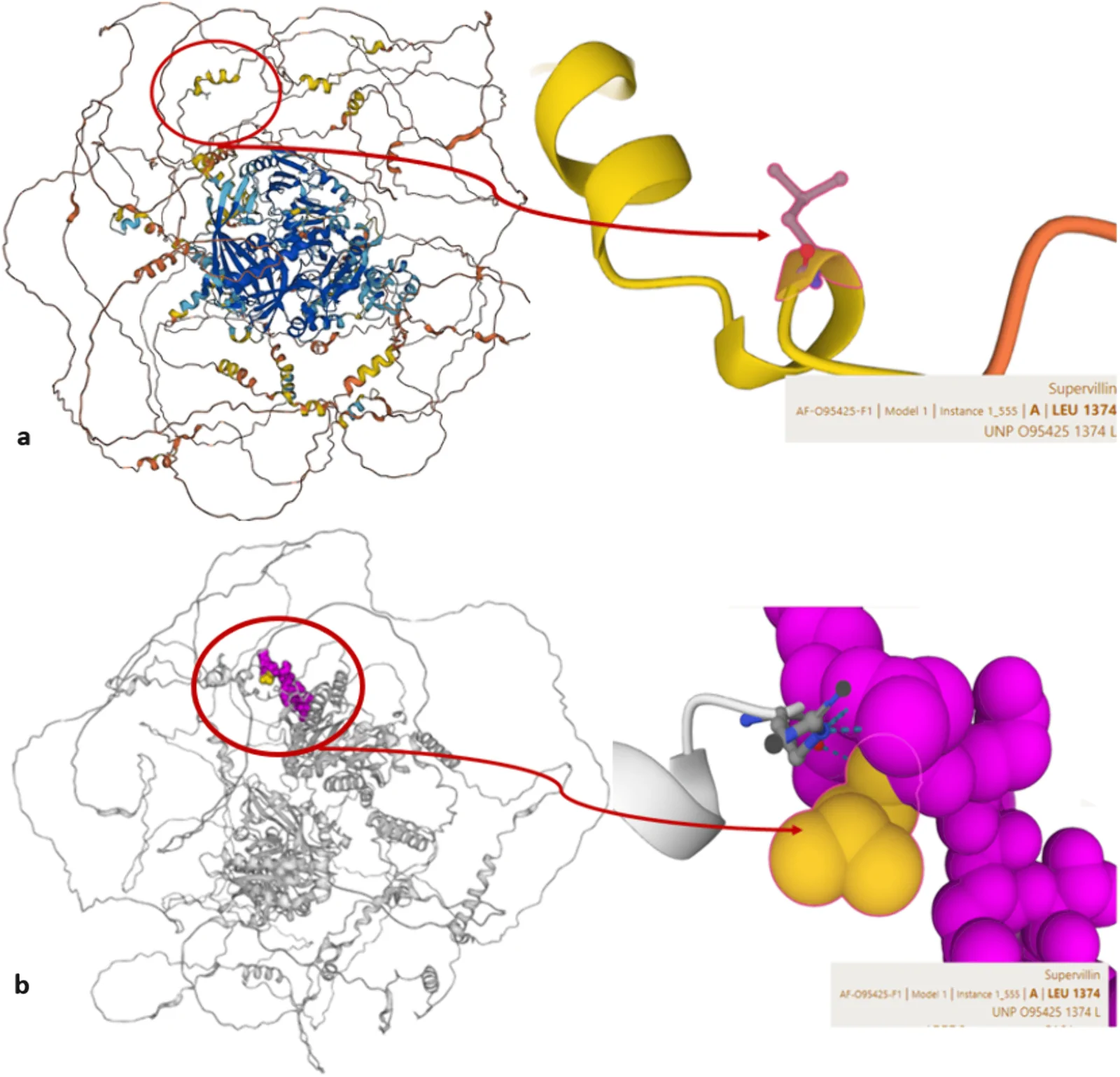
Graphical representation of rs199726033 (a) within the SVIL protein (b) located in PPI binding pocket (P55) pocket highlighted in pink, variant region within pocket is shown in yellow, amino acid residue changed from Leucine to Arginine at the 1374 amino acid position.

Additionally Varsite – EMBL database was utilized for the generation of a residue report for Leu1374Arg in the SVIL protein [44] The residue report gives useful information related to the possible impact of the variant on protein function, depicted visually in Fig 5. Variant localization shows its location within the Gelsolin repeat domain (Pfam: PF00626), a functionally important conserved protein domain as reflected by the dense cluster of tall red bars in the conservation track of Fig 5a. The domain coordinates were verified using data from the Uniport and Interpro databases [42,43]. The amino acid substitution is assumed to be likely deleterious as denoted by the CADD assessment of variant impact (purple denotes likely deleterious effect). Moreover, a substantial polarity shift consequent to replacement of Leucine (a non-polar, aliphatic residue with a hydrophobicity value +1.70) by Arginine (a positively charged, polar residue, highly hydrophilic at −1.01) is illustrated by the large charge gap in the Fauchère & Pliska hydrophobicity scale (Fig 5b &c). Pathogenicity assessment was also done utilizing the Alpha Missense heatmap prediction model [41], the L1374R substitution within the SVIL protein yielded a high pathogenicity likelihood score of 0.890 (Fig 8d). Furthermore, the VarSite database yielded a disease propensity score of 2.36, further supporting a likely pathogenic effect (Fig 8e).

**Fig 5:**
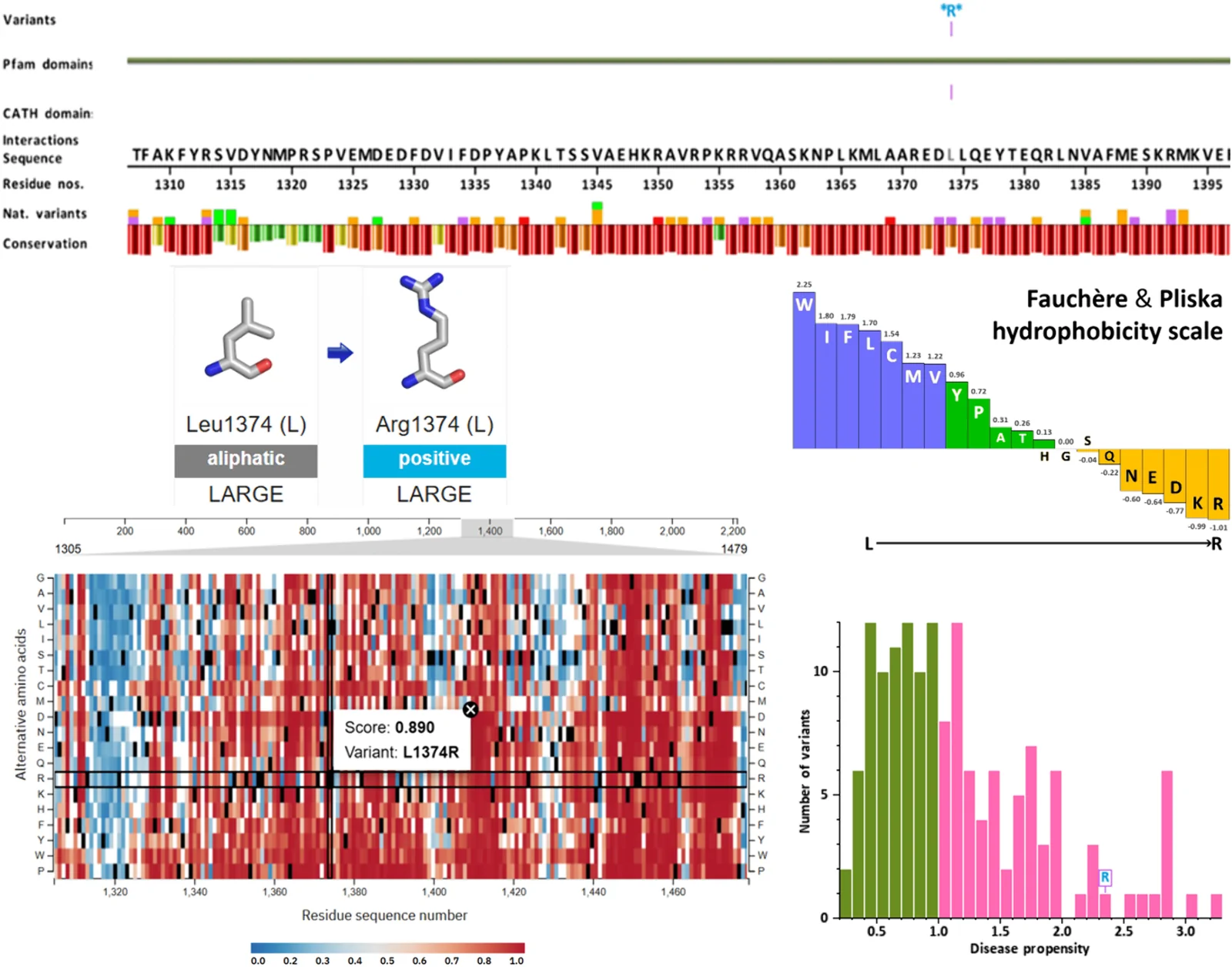
Residue Report showing Variant impact (a) The location of the Leu1374Arg variant (asterisked) in the SVIL protein is part of the Pfam A domain represented by the continuous green line. The red bars indicate that the variant lies in a highly conserved region (b) Structural representation of the Leu1374Arg mutation. Leucine (non-polar, aliphatic) is replaced by Arginine (positively charged, polar), introducing a significant change in side chain properties, represented by the purple coded CADD score indicating a likely deleterious impact of the missense mutation. (c) Bar graph representing amino acid hydrophobicity values according to the Fauchère & Pliska hydrophobicity scale. The plot indicates a notable polarity shift between Leucine (hydrophobic, + 1.25) to Arginine (hydrophilic, −1.01) that may alter the protein structural and functional properties. (d) Alpha Missense Pathogenicity Heatmap illustrates the pathogenicity likelihood for each possible amino acid substitution within the SVIL protein, pathogenicity likelihood scores are color coded, blue (0.0-0.34) indicate likely benign variants, grey (0.34-0.56) indicate uncertain effect and red (0.56-1) indicate likely pathogenic substitutions. The L1374R substitution marked on the heat map exhibits a pathogenic score of 0.890. (e) Histogram displaying the disease propensity scores for various amino acid substitutions. Green bars denote substitutions less frequently associated with disease while pink bars indicate high disease propensity. The L1374R variant, marked with a blue “R” demonstrated a high disease propensity score of 2.36, indicating a likely pathogenic effect.

An advanced algorithm-based tool the MutPred2, which quantifies the pathogenicity of amino acid substitutions by probabilistically modeling variant impact on specific protein structural and functional parameters was utilized to further investigate the mutational impact (Fig 6d). The L134R substitution returned a high MutPred pathogenicity score of 0.776, strongly indicative of a deleterious effect. The prediction highlighted a high probability of altered coiled coil regions, disruption at a disordered interface, and loss of sulfation at a nearby tyrosine residue (Y1378), these alterations may act as contributors to structural instability or impaired function.

**Fig 6:**
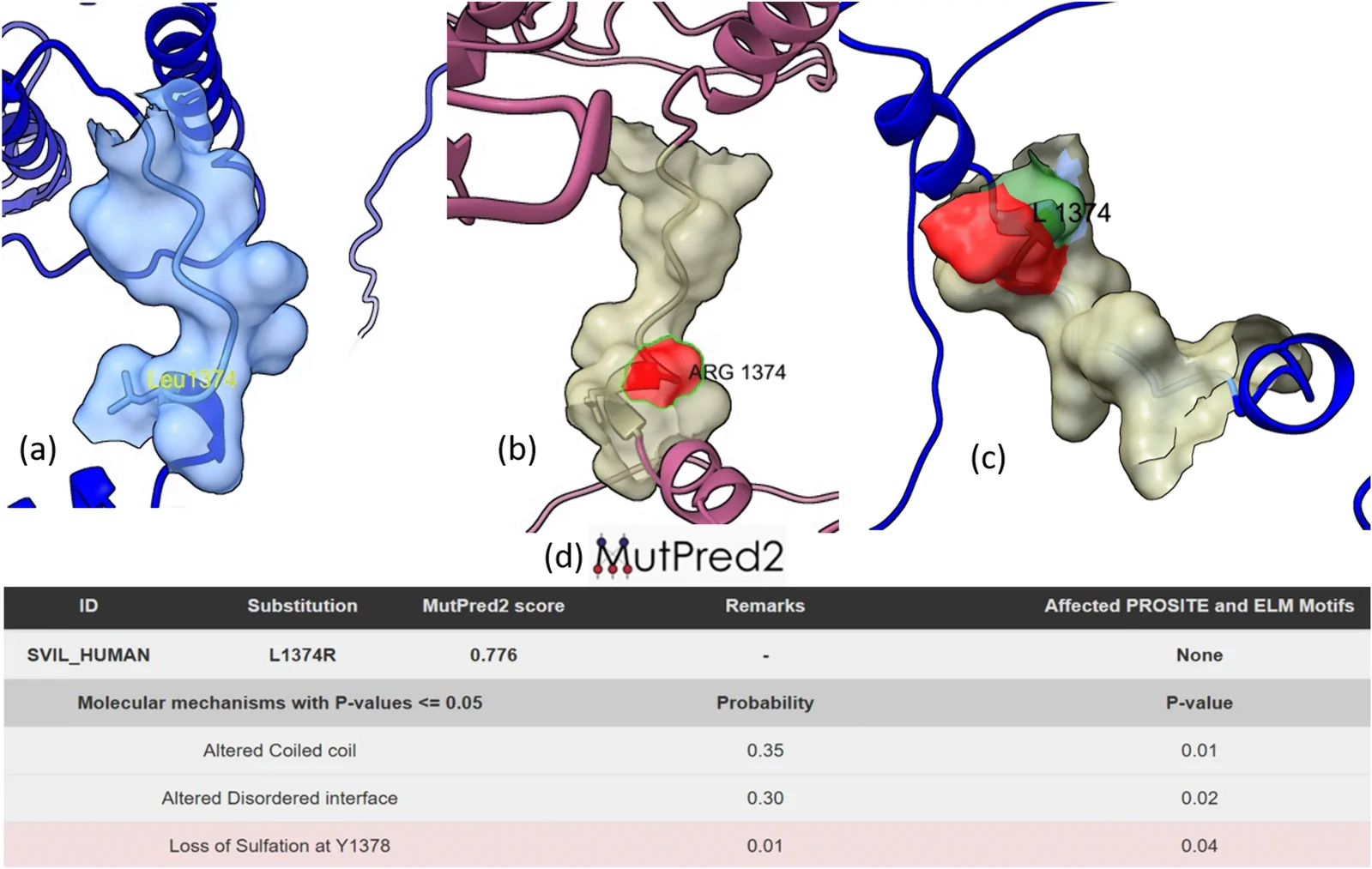
Graphical representation of the structural and functional impact of the L1374R mutation (a) Wild-type Leucine 1374 (Leu1374) located within its predicted binding pocket (P55). (b) The missense variant with Arginine 1374 (Arg1374) spatially oriented within the pocket. (c) Superimposition of wild-type (green) and mutant (red) models highlights local conformational shifts caused by the L1374R substitution. (d) MutPred2 prediction results for L1374R indicate a high pathogenicity score (0.776), with probable structural and functional consequences including disruption of coiled-coil regions, altered disordered interface, and loss of sulfation at nearby Tyrosine 1378 (Y1378).

### 3.5. Tissue expression, functional, and protein–protein interaction network of SVIL

Baseline SVIL expression across human tissues was assessed using the GTEX portal, a comprehensive resource of tissue and cell-specific gene expression and regulation [48]. Moderate expression levels were indicated for lung tissue while vascular and muscle tissues showed high expression as revealed by GTEX gene expression data as depicted in Fig 7.

**Fig 7:**
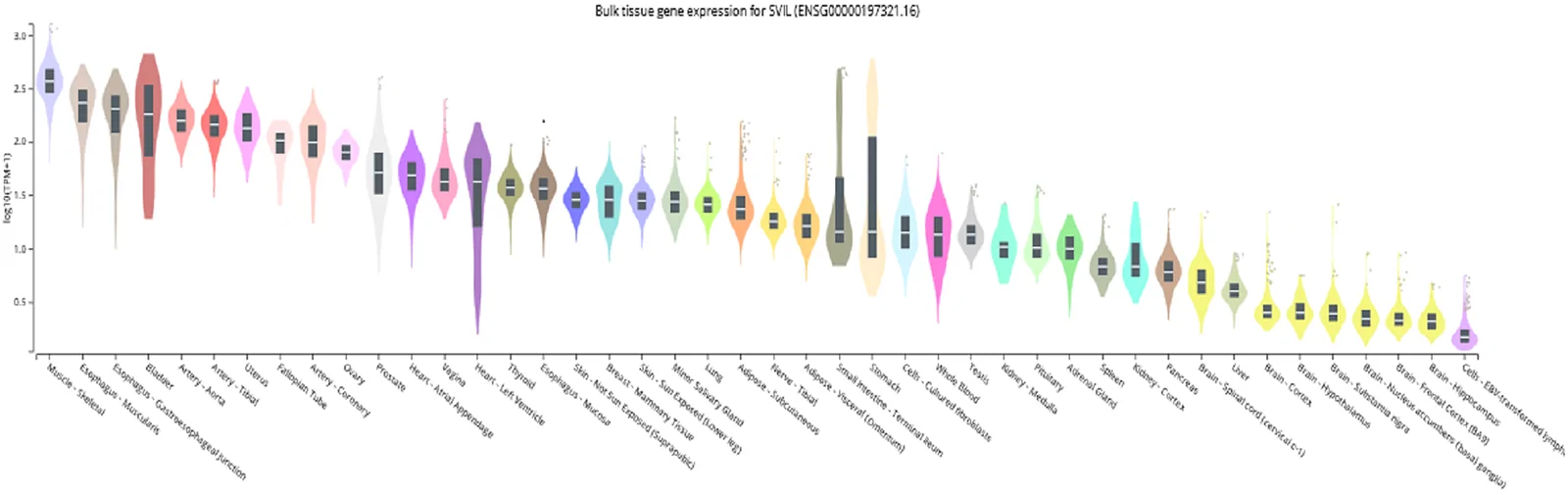
Baseline SVIL expression in body tissues retrieved from the GTEX portal. The expression level is represented on Y axis as median TPM (Transcripts per million), X-axis represents the different body tissues.

To explore SVIL’s molecular associations, GeneMANIA analysis was performed, revealing strong interactions with actin-binding and cytoskeletal regulatory proteins (Fig 8).

**Fig 8:**
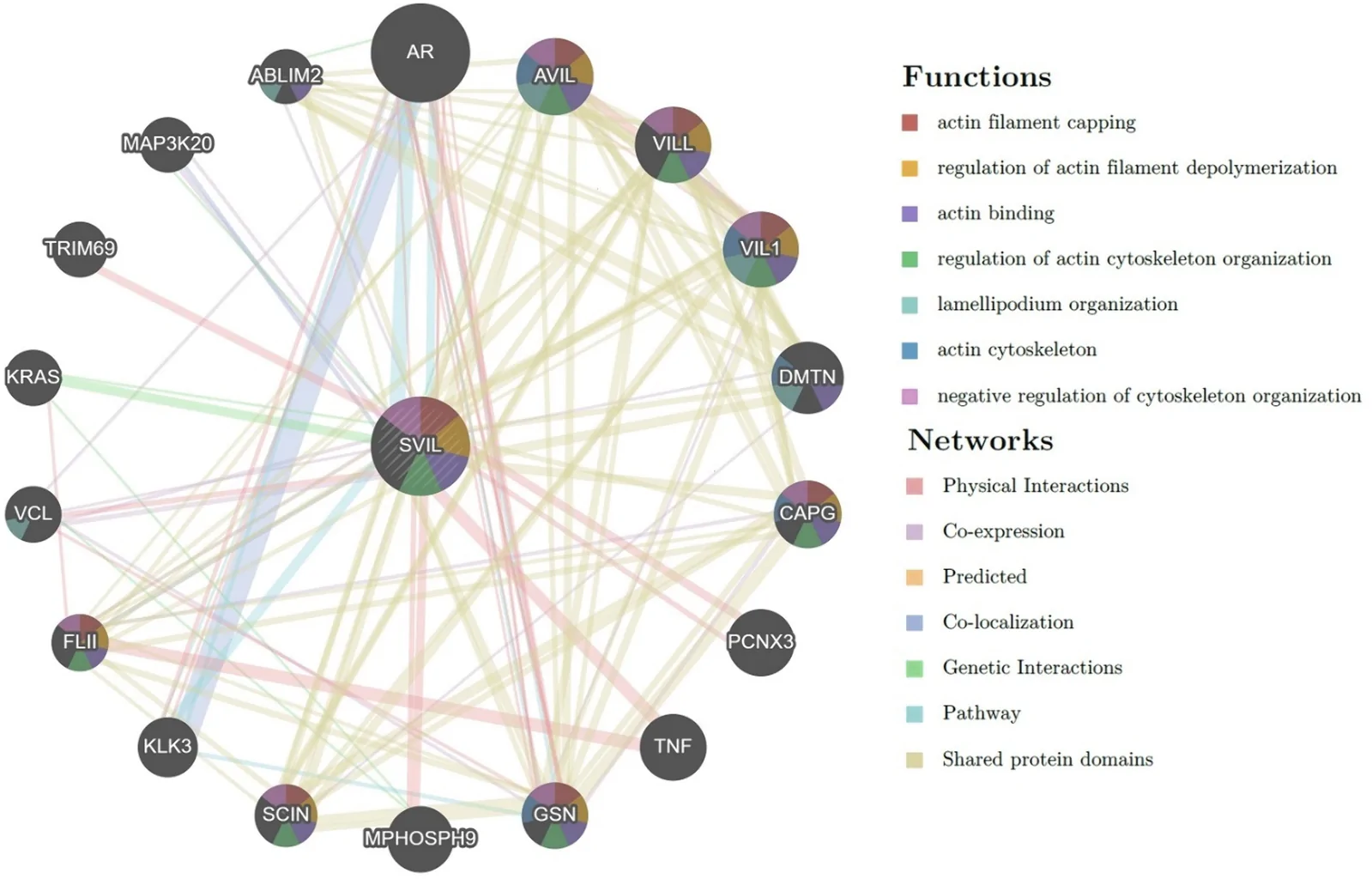
Gene association network of human SVIL gene. Nodes represent individual genes; the central gray dashed node represents the central SVIL gene. Colored links represent the different types of interactions among genes as indicated in key. SVIL shares protein domains with multiple genes as indicated by greenish grey lines. Light blue lines indicate SVIL, KLK3, and AR are involved in common molecular pathways. The size of the nodes represent the degree of interactions; the greatest interaction is found between AR and SVIL. Colors within the nodes indicate the common functional similarities of SVIL with other genes AVIL, VILL, SCIN, CAPG, FLII, VIL1, and GSN, regulating actin polymerization, actin binding, regulation of actin cytoskeleton organization, as indicated in the interactome key (Gene-gene interaction network constructed by Gene Mania) [46].

The SVIL protein’s interaction network was investigated using the IntAct web server, incorporating both human–human and virus–host interaction datasets (Fig 9). Notably, SVIL appeared within the SARS-CoV-2–human contactome, displaying documented interactions with several cytoskeletal and regulatory proteins, as well as viral proteins from SARS-CoV-2 and HPV.

**Fig 9:**
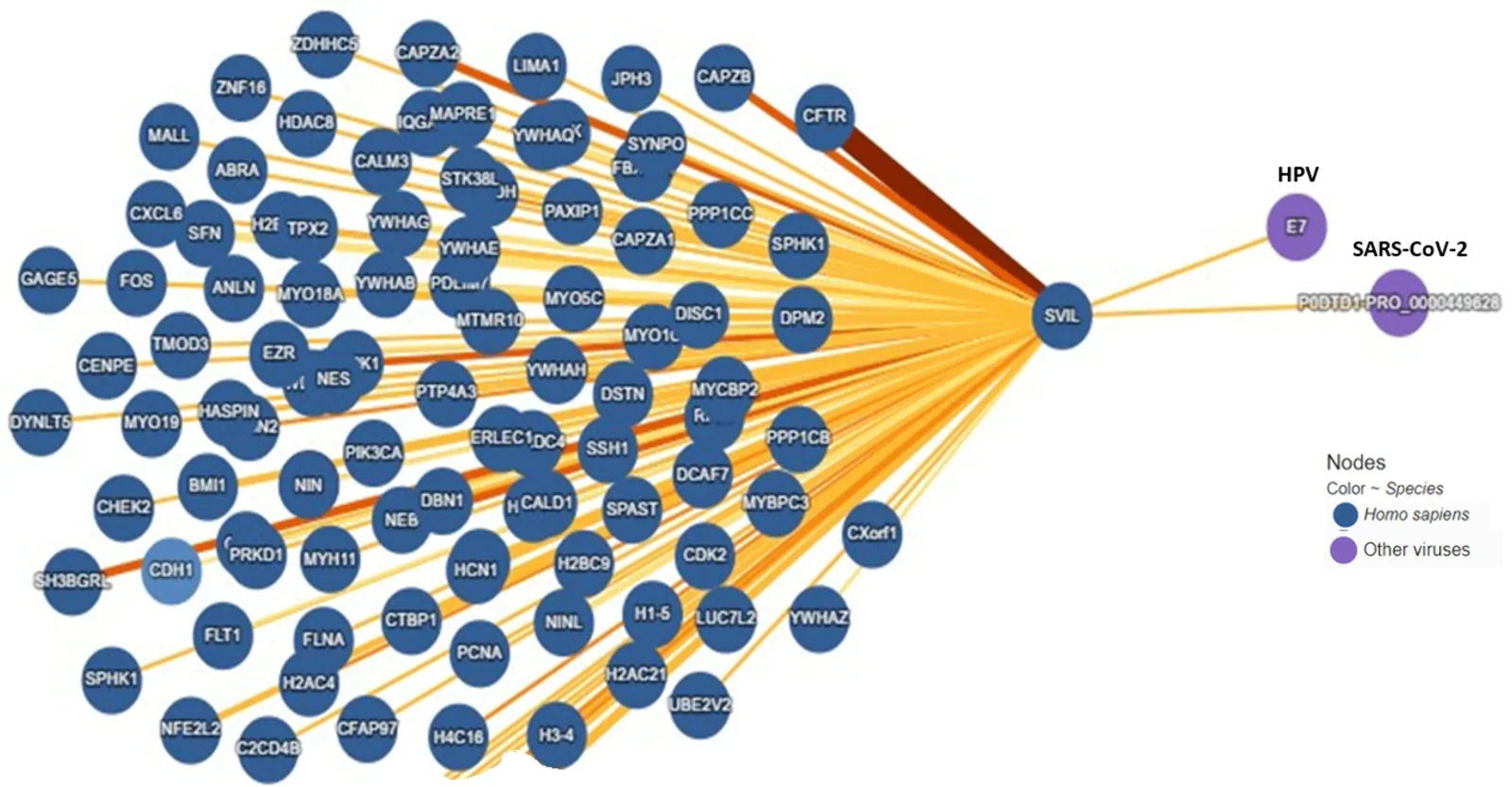
Protein-Protein interactome. Human SVIL protein interaction with other human proteins is indicated by blue nodes. SVIL interaction with viral proteins of HPV and SARS-CoV-2 is represented by purple nodes. Interaction strength is represented by the line thickness and color intensity (yellow to red). SVIL protein interactome generated by the IntAct database [49].

## 4. Discussion

The present study identifies a novel candidate genetic contributor to COVID-19 severity within the Pakistani population, implicating a rare SVIL variant (rs199726033, L1374R) in disease pathogenesis. SVIL(Supervillin) is a cytoskeletal regulatory protein that plays an active role in actin remodeling and membrane dynamics, processes frequently exploited by viruses during cellular infection. The structural and functional modeling of the L1374R substitution provides mechanistic evidence that this alteration may perturb critical protein–protein interactions, offering a plausible molecular link between host cytoskeletal remodeling and severe disease outcomes.

SVIL encodes a cytoskeleton-associated protein that regulates actin filament capping, depolymerization, and the formation of high-affinity linkage connections between the cytoskeleton and the plasma membrane [46,50]. As a member of the VILLIN protein-coding gene family (PTHR11977), which includes other non-motor actin-binding cytoskeletal proteins like Advillin, Villin and Gelsolin [51], SVIL localizes to the cell membrane, and other subcellular locations, predominantly the cytoskeleton, and the actin-rich cellular filopodia such as the invadopodia and podosomes (UniProt ID: 095425). These structures are particularly relevant to viral pathogenesis, as invadopodia promote extracellular matrix degradation, facilitating viral cell entry into host cells and metastatic cell invasion [52,53].

Cytoskeleton plays a vital role in many essential cell functions like endocytosis, transmission of different forces in the cell environment, and remodeling of different intracellular vacuoles in response to these forces [54]. The cytoskeleton is primarily composed of three main types of cytoskeletal polymers, including actin filaments (AFs), microtubules (MTs), and intermediate filaments (IFs) [55]. The most dynamic of these cytoskeletal proteins is actin, which is capable of bringing about structural reorganization promptly in response to internal or external stimuli and also influences the viral-host interactions [56]. Viruses have shown ability to surf to entry sites after binding to the host cell. Actin depolymerization which is one of the essential cell functions regulated by the SVIL gene is a key process involved in actin filaments rearrangement along the plasmalemma essential for facilitating viral cell entry [55]. After internalization of the virus and the subsequent processes of cell replication, the molecular rearrangement of actin filaments plays a crucial role [56,57]. Subplasmalemmal cortical actin accumulation was visualized in SARS-CoV-2-infected cells by electron microscopy, in a year 2020 study focusing on SARS-CoV-2-infected cells, suggesting the role of actin in virion entry, release, and transmission [58]. Research exploring the intricate involvement of cytoskeletal processes at different stages of SARS-CoV-2 pathogenesis has elucidated several mechanisms by which the virus exploits the host actin cytoskeleton. The viral infected cells undergo cytoskeletal modifications at the ultrastructural level in the form of filopodia and nanotube formation that promotes cell invasion and cell to cell transfer of SARS-CoV-2 [59,60]. Actin modulation is actively utilized to alter cortical actin resulting in reduced cell surface stiffness thereby promoting viral cell entry through the dephosphorylation and subsequent inactivation of ERM (ezrin/radixin/moesin) proteins which link the actin cytoskeleton to the plasma membrane [61]. Actin polymerization has also been identified as one of the key processes involved in SARS-CoV-2 spike protein-induced cell-cell fusion and syncytia formation which are multinucleated giant cell structures that propagate release of viral particles and trigger abnormal pulmonary inflammation [62]. Actin reorganization continues to play a critical role beyond the initial stages of viral infection. F-actin ring shaped nanostructures colocalize with viral M protein clusters and vesicles during the later stages of SARS-CoV-2 infection hijacking the intracellular cytoskeletal machinery through F-actin remodeling and filopodia formation to drive viral assembly, trafficking, and cell-to-cell transmission in pulmonary cells [63]. Additionally, Tunneling nanotubes (TNTs) and F-actin based membranous protrusions connecting distant cells are also formed serving as conduits for cell to cell viral spread, allowing the virus to bypass extracellular neutralizing antibodies and to disseminate efficiently within tissues [60]. Collectively, these observations establish the active involvement of actin cytoskeleton remodeling at different stages of viral pathogenesis. Recent reviews emphasize that understanding the SARS-CoV 2 interactions with the actin cytoskeleton is critical for developing novel antiviral therapies targeting actin driven viral pathogenesis [64].

The in silico analyses provide a plausible structural basis for the L1374R variant’s potential pathogenicity. The variant is localized within a highly conserved gelsolin repeat domain, a region critical for actin filament remodeling [47]. The critical nature of this particular protein domain is emphasized by the established link of Gelsolin (GSN) protein in COVID-19 pathophysiology. Significant changes in Plasma gelsolin (pGSN) levels are reported in critical COVID-19 cases. This finding correlates with the known role of Gelsolin in scavenging actin and regulation of immune responses in COVID-19 patients [65,66]. Reduced plasma gelsolin (pGSN) levels correlate with poor disease outcomes, including aggravated inflammatory responses, cytokine storm development, acute respiratory distress syndrome (ARDS), and organ failure [67,68]. Research is ongoing to study the potential therapeutic value of GSN in mitigating the key inflammatory and vascular pathophysiological events underlying severe COVID-19. Preclinical studies and preliminary clinical evidence from case reports and case series suggest that GSN supplementation may improve oxygenation, vascular permeability, and lung function in severe COVID-19 patients with ARDS [69–71]. These observations suggest a potential link of the gelsolin-actin interaction and the importance of this protein family in maintaining cytoskeletal and immune homeostasis. Moreover, structural modeling localized L1374R to a specific protein-protein interaction (PPI) binding pocket (P55) (Fig 4). The mutation causes a radical physicochemical shift, replacing a non-polar leucine with a positively charged, polar arginine, within a precise interaction site (Fig 5). The combination of these important factors explain the consistently deleterious pathogenicity scores for L1374R substitution. Furthermore, MutPred2 analysis raises probability of altered and disrupted coiled-coil motifs and indicates that the missense mutation abolishes a putative sulfation site at the nearby Y1378 position. Collectively, the pathogenicity predictions support a model whereby the L1374R variant impairs SVIL function by directly destabilizing protein-protein interactions essential for cytoskeletal regulation.

These findings are further contextualized by the gene’s expression profile. GTEx portal data reveals moderate SVIL expression in lungs, vascular and smooth muscle tissues [72] cell types frequently implicated in COVID-19 related pathophysiology. Recent literature consistently demonstrates that vascular pathology is not merely a secondary consequence but a key driver of COVID-19 severity and its long-term complications [73,74]. Notably, we have identified a genetic variant in one of the gelsolin repeat domains of the SVIL protein, a structural component central to actin filament regulation. In this context the established role of GSN protein, which can be regarded as a close functional analogue in maintaining vascular integrity, actin dynamics and immune modulation provide additional biologically plausible support for SVILs potential involvement in similar pathways.

The potential link between SVIL and severe COVID-19 pathophysiology is further reinforced by prior evidence of its role in vascular disorders. The SNP rs199726033 has previously been reported in cerebral aneurysm tissue, where downregulation of SVIL was proposed to disrupt actin regulation networks and contribute to vascular wall instability [75]. Experimental evidence from the same study demonstrated that SVIL knock down in vascular smooth muscle cells (vSMCs) caused overactivation of the Rho/ROCK pathway in turn leading to increased expression of ROCK which acts as a down-stream regulator of F-actin filaments. ROCK in turn accelerates the phenomenon of phenotypic switching of vSMCs moderated by its effect on actin polymerization [75]. Phenotypic switching of vSMCs refers to the morphological switching of cells between the contractile and synthetic forms [76]. It is a key process in the development of many vascular pathologies like hypertension, atherosclerosis and cardiovascular diseases [76,77]. Recent literature suggests the role of vSMCs phenotypic switching as one of the contributors to COVID-19 related vasculopathy and its complications [78]. It is noteworthy that significant links have been demonstrated between the Rho/ROCK pathway and COVID-19, particularly in lung pathology and potential therapeutic interventions. Multiple studies focusing on COVID-19 pathogenesis indicate aberrant ROCK activation caused by the overactivation of Rho/ROCK signaling pathway which increases inflammation, immune cell migration, and endothelial dysfunction, the key mechanisms associated with acute lung injury and other systemic effects [79,80]. Multiple studies have reported promising effects of Rho-kinase inhibitors as potential therapeutic agents for COVID-19–related pulmonary fibrosis, vascular dysfunction, and immune-mediated injury [81–83]. These benefits are thought to arise from inhibition of the ROCK pathway, which interacts with ACE2 and vascular endothelial growth factor (VEGF) signaling and has been shown to suppress SARS-CoV-2 infection and reduce viral replication [83]. Interestingly another genome-wide association study demonstrated significant links between SVIL variants and platelet function. Experimental evidence utilizing mouse knockout models and gene expression analysis suggest an inhibitory role for the SVIL gene in platelet adhesion and arterial thrombosis [84]. Collectively, these findings support a plausible link between SVIL dysregulation, Rho/ROCK pathway activation, platelet dysfunction and COVID-19–related vascular pathologies, consistent with the broader role of gelsolin-domain proteins in cytoskeletal remodeling.

In addition to its structural and cytoskeletal roles, SVIL has also been identified within the SARS-CoV-2–human protein interactome, suggesting possible involvement in host–virus interaction networks [85,86]. PPI data generated by the IntAct web server revealed SVIL proteins interactions with a myriad of human proteins including CFTR, in addition to the viral proteins of SARS-Cov-2 and HPV supporting its potential relevance in viral pathophysiology (Fig 9) [85,86]. The identification of a SVIL variant in the severe COVID-19 cohort further reinforces this connection, suggesting that genetic alterations in cytoskeletal regulatory proteins may modulate disease severity through effects on, actin dynamics, vascular integrity and inflammatory signaling. In the broader context, these findings highlight the potential relevance of host cytoskeletal gene variants in shaping susceptibility to viral pathogenesis and outcomes across diverse viral infections.

In addition to SVIL, five other missense variants located within GGN, KLK14, NRG3, CHST6, CCDC88B were identified in the discovery cohort, meeting the variant prioritization and recurrence criteria. Although the carrier frequencies of these variants were lower (3–4/12) as compared to SVIL (7/12), the co-occurrence of these missense, deleterious variants within a subset of severe disease patients (P3, P11, P10, P12) suggests a potential cumulative genetic risk (Fig 2b). Among these NRG3 is of particular relevance in the context of its presence in severe disease patients, as it has been identified as a candidate risk mediator of COVID-19 severity and associated polymyositis in a recent study [87]. NRG3 activates ErbB3/ErbB4 receptors and downstream PI3K/Akt and Ras/MAPK pathways, regulating cell growth, survival, and tissue repair. The ErbB receptors regulate viral entry through direct binding to the SARS-CoV-2 S1 protein, while NRG3/ ErB4 signaling has also been linked to muscle pathology. Additionally, the identification of CCDC88B and KLK14 suggests a genetic basis of immune dysregulation in severe Covid-19. CCDC88B has established roles in T-cell function and dendritic-cell migration, whereas KLK14 can modulate complement-related innate immune responses [88,89]. Furthermore, CCDC88B shows transcriptional relevance as one of the common differentially expressed genes (DEGs) at the intersection of COVID-19 and metabolic unhealthy obesity [90]. However, these associations are indirect and are not evidence of gene-specific involvement in SARS-CoV-2 infection. Collectively, the findings support a hypothesis in which NRG3 and other deleterious variants mark convergent immune related pathogenesis contributing to disease severity. Nevertheless given the small discovery cohort, these observations are exploratory and require validation in larger independent cohorts.

## 5. Limitations

Since the study was performed in Pakistan which is a low to medium income country, the sample size was limited owing to high sequencing costs and financial constraints. Generalizability of the findings may thus be restricted. However, this is a common limitation in initial exploratory WES studies especially those focusing on underrepresented populations. Furthermore, we acknowledge that WES primarily targets coding regions and GWAS or whole-genome sequencing could better explore intronic regions and regulatory variants. Nevertheless, WES was better suited in the context of the primary aim of of identifying low frequency variants in the current study. Future large multicentric studies are warranted to validate and extend the findings. Additionally, individual level SARS-CoV-2 genotyping was not performed; however, the sampling period coincides with the Omicron variant predominance in Pakistan and potential variant dependent effects on disease severity cannot be excluded.

## 6. Conclusion

The identification of the rs199726033 missense variant in SVIL implicates this locus as a potential candidate for COVID-19 severity. This association is biologically plausible given SVIL’s role in actin cytoskeleton regulation, a process involved in viral cell entry, vasculopathy and immune regulation. Thus, we conclude that genetic variation in SVIL could influence host pathways relevant to severe viral infection.

## Supporting information

S1 Fig SNP density plot showing chromosomal distribution of variants. SNP density plot shows number of SNPs across chromosomes in 1 MB (mega base) genomic region. The color gradient, from green (low) to red (high), reflects increasing SNP counts.
(TIF)

S2 Fig IGV Visualization of the SVIL variant (rs199726033) in Discovery Cohort. IGV visualization of the SVIL variant (rs199726033) in the discovery cohort. Integrative Genomic Viewer (IGV) display showing the WES alignment at chr10:29,490,918 (hg38) for multiple severe disease patients. The variant (A > C, p. Leu1374Arg) is highlighted. The. bam alignment track displays clean, symmetric read coverage across both strands at the variant locus, with no evidence of strand bias, mapping gaps, or low quality read end clustering. The VCF tracks confirm consistent variant calling across multiple samples.
(TIF)

S3 Fig Amino acid sequence alignment. Amino acid sequence alignment of the 420 bp *SVIL* exon 22 region across sequenced samples. Alignment was generated using *Jalview,* following reverse complementation of the nucleotide sequences to adjust for *SVIL* location on the reverse strand. The position of the L1374R mutation is highlighted in red. Conservation and quality bars below indicate the level of residue conservation across the cohort. The mutation lies within a moderately conserved motif, suggesting potential structural/functional relevance.
(TIF)

S1 Table Demographic., Clinical and Lab parameters of study cohort. (XLSX)

S2 Table Clinical and Genotypic characteristics of discovery cohort patients. (XLSX)

S3 Table Prioritized variants in the 12 discovery cohort patients. (XLSX)

S4 Table Association of SVIL Gene Variants with Severe COVID-19 in the Validation Cohort. (XLSX)

S5 Table Annotation and functional characterization of *SVIL* variants across samples in the Sanger validation cohort. (XLSX)

## Abbreviation:

ACE2: Angiotensin-Converting Enzyme 2
ACMG: American College of Medical Genetics and Genomics
AR: Androgen Receptor
AVIL: Advillin
BAM: Binary Alignment/Map file
BWA: Burrows–Wheeler Aligner
BWA-MEM: Burrows–Wheeler Aligner Maximal Exact Matches algorithm
CADD: Combined Annotation Dependent Depletion
CAPG: Capping Actin Protein, Gelsolin-Like
CCDC88B: Coiled-Coil Domain Containing 88B
CCR2: C-C Motif Chemokine Receptor 2
CDC: Centers for Disease Control and Prevention
CHST6: Carbohydrate Sulfotransferase 6
COVID-19: Coronavirus Disease 2019
CRP: C-Reactive Protein
CTAB: Cetyltrimethylammonium Bromide
CXR: Chest X-Ray
DIN: DNA Integrity Number
DNA: Deoxyribonucleic Acid
DPP9: Dipeptidyl Peptidase 9
EDTA: Ethylenediaminetetraacetic Acid
EMBL-EBI: European Molecular Biology Laboratory – European Bioinformatics Institute
FLII: Flightless I Actin Remodeling Protein
GATK: Genome Analysis Toolkit
gDNA: Genomic DNA
GGN: Gametogenetin
gnomAD: Genome Aggregation Database
GRCh38: Genome Reference Consortium Human Build 38
GSN: Gelsolin
GTEx: Genotype-Tissue Expression (portal)
GWAS: Genome-Wide Association Study
HPV: Human Papillomavirus
HRCT: High-Resolution Computed Tomography
ICU: Intensive Care Unit
IFNAR2: Interferon Alpha and Beta Receptor Subunit 2
KLK3: Kallikrein-Related Peptidase 3
KLK14: Kallikrein-Related Peptidase 14
L1374R: Leucine-to-Arginine substitution at amino acid position 1374
LZTFL1: Leucine Zipper Transcription Factor-Like Protein 1
MAF: Minor Allele Frequency
NRG3: Neuregulin 3
PCR: Polymerase Chain Reaction
PI3K/Akt: Phosphoinositide 3-kinase / Protein Kinase B
Ras/MAPK: Rat Sarcoma/ Mitogen-Activated Protein Kinase
Rho-ROCK: Ras Homolog Family–Rho-Associated Coiled-Coil Containing Protein Kinase pathway
SARS-CoV-2: Severe Acute Respiratory Syndrome Coronavirus 2
SIFT: Sorting Intolerant from Tolerant
SNP: Single Nucleotide Polymorphism
SPSS: Statistical Package for the Social Sciences
SVIL: Supervillin
TMPRSS2: Transmembrane Protease Serine 2
TYK2: Tyrosine Kinase 2
VCF: Variant Call Format
VEP: Variant Effect Predictor
VIL1: Villin 1
VILL: Villin-Like Protein
WES: Whole-Exome Sequencing
WHO: World Health Organization
