# Supplementary material for: Whole-exome sequencing identifies a missense variant in the cytoskeletal gene SVIL implicating actin remodeling in COVID-19 severity in a Pakistani cohort
Source: PLoS One. 2026 Oct 12;21(10):e0360429. doi: 10.1371/journal.pone.0360429 (PMC13657318; doi:10.1371/journal.pone.0360429)
Supplement: Supplementary file 1 [file pone.0360429.s010.docx]

**Response to Reviewer:**

We are grateful to the reviewer for the valuable feedback. We have carefully considered all comments and have revised the manuscript accordingly.

Point-by-point responses are provided below, with changes highlighted in the revised version.

**Comment 1:** *What ails WES when compared to a simple GWAS, wherein they could have possibly gotten some more pathogenic and noncoding variants*

**Response:** We thank the reviewer for this important observation. The choice between WES and GWAS depends on the primary objective of the study. Whole-exome sequencing (WES) directly captures protein-coding variation across the allelic spectrum, whereas genome-wide association studies (GWAS) rely on genotyping arrays and imputation that are poorly suited to rare and low-frequency variants particularly in South Asian populations where imputation reference panels are limited(1). The two approaches are best viewed as complementary, GWAS excels at common variant discovery, while WES is the primary tool for rare coding variant association and pathogenic variant identification which was the primary objective of the current study(2, 3).

We acknowledge that GWAS could have captured additional noncoding regulatory variants and suggest that future genome-wide approaches may further explore these regions. This point has been added to the limitation section in the revised manuscript (page 14, lines 490-492)

Nonetheless, our WES approach successfully identified multiple prioritized pathogenic variants in the discovery cohort of severe disease patients, including a recurring panel involving *SVIL, GGN*, *NRG3* and *KLK14*, supporting the utility of WES for exploratory variant discovery in understudied populations. To address the reviewers concern regarding other pathogenic variants, we have replaced Figure 2b with an updated heatmap visualizing the distribution of all six prioritized variants across the discovery cohort. The discussion section has been updated accordingly to elaborate on the significance of these co-occurring pathogenic variants in severe patients (page 13, 14, lines 469-485).

**Comment 2:** *While the variant was NOT validated using downstream approaches, they could have justified it using IGV browser*

**Response:** We appreciate the reviewer’s suggestion to display the genomic data within the Integrative Genomic Viewer (IGV) to showcase the raw alignments quality. Accordingly, we have added Supporting FigureS2 to the manuscript. The methods (page 4, lines 147-148) and results section (page 7, lines 242-243) have been updated accordingly and additions are highlighted.

As visualized via IGV the WES discovery cohort samples VCF tracks confirm that the variant was consistently called at chr10:29,490,918 (hg38) across multiple samples. Inspection of the raw WES alignments (.bam) files confirmed clean, symmetric read coverage across both strands with no evidence of strand bias. Absence of mapping gaps and low quality read end clustering were confirmed. These in silico observations are consistent with our sanger sequencing validation. We thank the reviewer for prompting this improvement which has helped us improve the depth and transparency of our genomic data visualization.

**Comment 3:** *What is the relative importance of cytoskeletal processes during COVID times is NOT justified*

**Response:** We thank the reviewer for this important observation. To address this comment, we have added a detailed paragraph in the discussion section (page 12, lines 387-406) summarizing the current literature on the relevance of cytoskeletal processes in different stages of COVID-19 pathogenesis. This added text establishes the mechanistic basis for SVILs potential role in modulating COVID-19 severity through its role in regulation of actin dynamics.

**Comment 4:** *The age groups and severity within specific time period and type of SARS-COV-2 variant of concern could have been included*
**Response 4:** We are grateful for the opportunity to clarify this important point. Regarding age and its relevance with severity, the results section includes a statement on page 6, line 215,216 describing that severe patients were significantly older (median 73 years contrary to 56 years, p <0.001). This is consistent with published data from Pakistan indicating that 60 to 79 years were the most common ICU admissions during the pandemic(4).

Regarding the specific time period and its relevance to age and severity, the sampling period of our study coincides with the fifth wave of Covid-19 in Pakistan during which the Omicron variant was prevalent. A recent multicenter study documenting data from the Pakistan Registry of Intensive CarE (PRICE) notes that the Omicron variant was associated with lower mortality as compared to its preceding variants of concern(4). The study also documents an increased proportion of older individuals (>80 years) being admitted during the fifth wave (18.6%) as compared to the fourth wave (8.9%), suggesting that while older individuals continued to be admitted during the Omicron predominant wave, the variants lower virulence may have moderated disease severity. We have added this context to the Limitations section of the revised manuscript (page 14, line 493-495).

**Minor comments**
*L38: Pl correct was to WERE
l232: pl remove comma after 94%
Limitations can move before conclusions
L160: in silico must be separate and preferably italicized*

**Response to minor comments:** Corrections made to the revised manuscript.

**References:**

1.	Sun BB, Kurki MI, Foley CN, Mechakra A, Chen C-Y, Marshall E, et al. Genetic associations of protein-coding variants in human disease. Nature biotechnology.603(7899):95-102 <https://doi.org/10.1038/s41586-022-04394-w(2022>).

2.	Kamenarova K, Kachakova-Yordanova D, Baymakova M, Georgiev M, Mihova K, Petkova V, et al. Rare host variants in ciliary expressed genes contribute to COVID-19 severity in Bulgarian patients. Scientific Reports.14(1):19487 <https://doi.org/10.1038/s41598-024-70514-3(2024>).

3.	Mir R, Ullah MF, Elfaki I, Alanazi MA, Algehainy NA, Altemani FH, et al. Whole-exome sequencing reveals rare genetic variants in saudi covid-19 patients with extreme phenotypes. Viruses.17(9):1198 <https://doi.org/10.3390/v17091198(2025>).

4.	Khan A, Nayeem A, White N, Siddiqui A, Hussaini A, 22 CTRSKCUI, et al. Critical care trends across five waves of COVID-19 in Pakistan: A multi-center observational study from the Pakistan Registry of Intensive CarE (PRICE).26(1):803 2026).
