# Supplementary material for: Whole-exome sequencing identifies a missense variant in the cytoskeletal gene SVIL implicating actin remodeling in COVID-19 severity in a Pakistani cohort
Source: PLoS One. 2026 Oct 12;21(10):e0360429. doi: 10.1371/journal.pone.0360429 (PMC13657318; doi:10.1371/journal.pone.0360429)
Supplement: Peer review history

# Peer review: Decision Letter 0

**DOI:** 10.1371/journal.pone.0360429.r001

**Authors:** Flora De Conto

14 Jul 2026

Dear Dr. Khatoon,

Thank you for submitting your manuscript to PLOS ONE. After careful consideration, we feel that it has merit but does not fully meet PLOS ONE’s publication criteria as it currently stands. Therefore, we invite you to submit a revised version of the manuscript that addresses the points raised during the review process.

==============================

**ACADEMIC EDITOR:**  The submitted article addresses a topic of interest that, to date, has been insufficiently explored. The cellular cytoskeleton mediates both viral infection and the activation of host defenses. Viruses possess the ability to co-opt cellular functions for their own benefit. The article raises several critical issues that need to be promptly addressed in accordance with the reviewer's recommendations.

==============================

Please submit your revised manuscript by Sep 12 2026 11:59PM. If you will need more time than this to complete your revisions, please reply to this message or contact the journal office at plosone@plos.org. When you're ready to submit your revision, log on to https://www.editorialmanager.com/pone/ and select the 'Submissions Needing Revision' folder to locate your manuscript file.

- A letter that responds to each point raised by the academic editor and reviewer(s). You should upload this letter as a separate file labeled 'Response to Reviewers'.
- A marked-up copy of your manuscript that highlights changes made to the original version. You should upload this as a separate file labeled 'Revised Manuscript with Track Changes'.
- An unmarked version of your revised paper without tracked changes. You should upload this as a separate file labeled 'Manuscript'.

If you would like to make changes to your financial disclosure, please include your updated statement in your cover letter. Guidelines for resubmitting your figure files are available below the reviewer comments at the end of this letter.

If applicable, we recommend that you deposit your laboratory protocols in protocols.io to enhance the reproducibility of your results. Protocols.io assigns your protocol its own identifier (DOI) so that it can be cited independently in the future. For instructions see: https://journals.plos.org/plosone/s/submission-guidelines#loc-laboratory-protocols. Additionally, PLOS ONE offers an option for publishing peer-reviewed Lab Protocol articles, which describe protocols hosted on protocols.io. Read more information on sharing protocols at https://plos.org/protocols?utm_medium=editorial-email&utm_source=authorletters&utm_campaign=protocols.

As the corresponding author, your ORCID iD is verified in the submission system and will appear in the published article. PLOS supports the use of ORCID, and we encourage all coauthors to register for an ORCID iD and use it as well. Please encourage your coauthors to verify their ORCID iD within the submission system before final acceptance, as unverified ORCID iDs will not appear in the published article. *Only*  the individual author can complete the verification step; PLOS staff *cannot*  verify ORCID iDs on behalf of authors.

We look forward to receiving your revised manuscript.

Kind regards,

Flora De Conto, Ph.D.

Academic Editor

PLOS One

Journal Requirements:

When submitting your revision, we need you to address these additional requirements.

1. Please ensure that your manuscript meets PLOS ONE's style requirements, including those for file naming. The PLOS ONE style templates can be found at

https://journals.plos.org/plosone/s/file?id=wjVg/PLOSOne_formatting_sample_main_body.pdf and https://journals.plos.org/plosone/s/file?id=ba62/PLOSOne_formatting_sample_title_authors_affiliations.pdf

2. The ethical approval number(s) listed in the manuscript and/or submission metadata does not match the approval number on the ethical approval document you provided. Please ensure that all approval numbers are correct.

3. Please note that PLOS One has specific guidelines on code sharing for submissions in which author-generated code underpins the findings in the manuscript. In these cases, we expect all author-generated code to be made available without restrictions upon publication of the work. Please review our guidelines at https://journals.plos.org/plosone/s/materials-and-software-sharing#loc-sharing-code and ensure that your code is shared in a way that follows best practice and facilitates reproducibility and reuse.

4. Your ethics statement should only appear in the Methods section of your manuscript. If your ethics statement is written in any section besides the Methods, please move it to the Methods section and delete it from any other section. Please ensure that your ethics statement is included in your manuscript, as the ethics statement entered into the online submission form will not be published alongside your manuscript.

5. Thank you for stating the following financial disclosure:

Higher Education Commission, HEC, Pakistan (Ref No. 2015891/NRPU/R&D/HEC/2021 2021)

Please state what role the funders took in the study. If the funders had no role, please state: "The funders had no role in study design, data collection and analysis, decision to publish, or preparation of the manuscript."

If this statement is not correct you must amend it as needed.

Please include this amended Role of Funder statement in your cover letter; we will change the online submission form on your behalf.

6. Please include captions for your Supporting Information files at the end of your manuscript, and update any in-text citations to match accordingly. Please see our Supporting Information guidelines for more information: http://journals.plos.org/plosone/s/supporting-information.

7. Please ensure that you refer to Figure S1 in your text as, if accepted, production will need this reference to link the reader to the figure.

8. If the reviewer comments include a recommendation to cite specific previously published works, please review and evaluate these publications to determine whether they are relevant and should be cited. There is no requirement to cite these works unless the editor has indicated otherwise.

[Note: HTML markup is below. Please do not edit.]

Reviewers' comments:

Reviewer's Responses to Questions

**Comments to the Author**

1. Is the manuscript technically sound, and do the data support the conclusions?

Reviewer #1: Yes

**********

2. Has the statistical analysis been performed appropriately and rigorously?

Reviewer #1: Yes

**********

3. Have the authors made all data underlying the findings in their manuscript fully available?

The PLOS Data policy

Reviewer #1: Yes

**********

4. Is the manuscript presented in an intelligible fashion and written in standard English?

Reviewer #1: Yes

**********

Reviewer #1: Gist/Summary: The authorsinvestigated host genetic associations with disease severity and their molecular basis in COVID-19 patients of South Asian origin, all coming from Pakistan wherein the patients were stratified by clinical and radiographic severity. They further performed whole-exome sequencing(WES) and high risk SNPs were taken by VEP through GATK calling and SIFT/CADD downstream annotations. The authors report Supervillin (SVIL) gene missense variant rs199726033 showing the highest recurrence in the discovery cohort, with a minor allele frequency to be significant which suggests clonal pathogenesis attributed to host cytoskeletal processes during COVID times.

The MS is generally written well, but I have a few questions

What ails WES when compared to a simple GWAS, wherein they could have possibly gotten some more pathogenic and noncoding variants

While the variant wa sNOT validated using downstream approaches, they could have justified it using IGV browser

What is the relative importance of cytoskeletal processes during COVID times is NOT justified

The age groups anbd severity within specific time period and type of SARS-COV-2 variant of concern could have been included

Minor but essential

L38: Pl correct was to WERE

l232: pl remove comma after 94%

Limitations canmove before conclusions

L160: in silico must be separate and preferably italcized

**********

what does this mean?). If published, this will include your full peer review and any attached files.

If you choose “no”, your identity will remain anonymous but your review may still be made public.

**Do you want your identity to be public for this peer review?**  For information about this choice, including consent withdrawal, please see our Privacy Policy

Reviewer #1: **Yes:**  Prashanth N Suravajhala

**********

[NOTE: If reviewer comments were submitted as an attachment file, they will be attached to this email and accessible via the submission site. Please log into your account, locate the manuscript record, and check for the action link "View Attachments". If this link does not appear, there are no attachment files.]

To ensure your figures meet our technical requirements, please review our figure guidelines: https://journals.plos.org/plosone/s/figures

You may also use PLOS’s free figure tool, NAAS, to help you prepare publication quality figures: https://journals.plos.org/plosone/s/figures#loc-tools-for-figure-preparation.

NAAS will assess whether your figures meet our technical requirements by comparing each figure against our figure specifications.

# Author comment: Author response to Decision Letter 1

**DOI:** 10.1371/journal.pone.0360429.r002

22 Sep 2026

Response to Reviewer:

We are grateful to the reviewer for the valuable feedback. We have carefully considered all comments and have revised the manuscript accordingly.

Point-by-point responses are provided below, with changes highlighted in the revised version.

Comment 1: What ails WES when compared to a simple GWAS, wherein they could have possibly gotten some more pathogenic and noncoding variants

Response: We thank the reviewer for this important observation. The choice between WES and GWAS depends on the primary objective of the study. Whole-exome sequencing (WES) directly captures protein-coding variation across the allelic spectrum, whereas genome-wide association studies (GWAS) rely on genotyping arrays and imputation that are poorly suited to rare and low-frequency variants particularly in South Asian populations where imputation reference panels are limited(1). The two approaches are best viewed as complementary, GWAS excels at common variant discovery, while WES is the primary tool for rare coding variant association and pathogenic variant identification which was the primary objective of the current study(2, 3).

We acknowledge that GWAS could have captured additional noncoding regulatory variants and suggest that future genome-wide approaches may further explore these regions. This point has been added to the limitation section in the revised manuscript (page 14, lines 490-492)

Nonetheless, our WES approach successfully identified multiple prioritized pathogenic variants in the discovery cohort of severe disease patients, including a recurring panel involving SVIL, GGN, NRG3 and KLK14, supporting the utility of WES for exploratory variant discovery in understudied populations. To address the reviewers concern regarding other pathogenic variants, we have replaced Figure 2b with an updated heatmap visualizing the distribution of all six prioritized variants across the discovery cohort. The discussion section has been updated accordingly to elaborate on the significance of these co-occurring pathogenic variants in severe patients (page 13, 14, lines 469-485).

Comment 2: While the variant was NOT validated using downstream approaches, they could have justified it using IGV browser

Response: We appreciate the reviewer’s suggestion to display the genomic data within the Integrative Genomic Viewer (IGV) to showcase the raw alignments quality. Accordingly, we have added Supporting FigureS2 to the manuscript. The methods (page 4, lines 147-148) and results section (page 7, lines 242-243) have been updated accordingly and additions are highlighted.

As visualized via IGV the WES discovery cohort samples VCF tracks confirm that the variant was consistently called at chr10:29,490,918 (hg38) across multiple samples. Inspection of the raw WES alignments (.bam) files confirmed clean, symmetric read coverage across both strands with no evidence of strand bias. Absence of mapping gaps and low quality read end clustering were confirmed. These in silico observations are consistent with our sanger sequencing validation. We thank the reviewer for prompting this improvement which has helped us improve the depth and transparency of our genomic data visualization.

Comment 3: What is the relative importance of cytoskeletal processes during COVID times is NOT justified

Response: We thank the reviewer for this important observation. To address this comment, we have added a detailed paragraph in the discussion section (page 12, lines 387-406) summarizing the current literature on the relevance of cytoskeletal processes in different stages of COVID-19 pathogenesis. This added text establishes the mechanistic basis for SVILs potential role in modulating COVID-19 severity through its role in regulation of actin dynamics.

Comment 4: The age groups and severity within specific time period and type of SARS-COV-2 variant of concern could have been included

Response 4: We are grateful for the opportunity to clarify this important point. Regarding age and its relevance with severity, the results section includes a statement on page 6, line 215,216 describing that severe patients were significantly older (median 73 years contrary to 56 years, p <0.001). This is consistent with published data from Pakistan indicating that 60 to 79 years were the most common ICU admissions during the pandemic(4).

Regarding the specific time period and its relevance to age and severity, the sampling period of our study coincides with the fifth wave of Covid-19 in Pakistan during which the Omicron variant was prevalent. A recent multicenter study documenting data from the Pakistan Registry of Intensive CarE (PRICE) notes that the Omicron variant was associated with lower mortality as compared to its preceding variants of concern(4). The study also documents an increased proportion of older individuals (>80 years) being admitted during the fifth wave (18.6%) as compared to the fourth wave (8.9%), suggesting that while older individuals continued to be admitted during the Omicron predominant wave, the variants lower virulence may have moderated disease severity. We have added this context to the Limitations section of the revised manuscript (page 14, line 493-495).

Minor comments

L38: Pl correct was to WERE

l232: pl remove comma after 94%

Limitations can move before conclusions

L160: in silico must be separate and preferably italicized

Response to minor comments: Corrections made to the revised manuscript.

References:

1. Sun BB, Kurki MI, Foley CN, Mechakra A, Chen C-Y, Marshall E, et al. Genetic associations of protein-coding variants in human disease. Nature biotechnology.603(7899):95-102 https://doi.org/10.1038/s41586-022-04394-w(2022).

2. Kamenarova K, Kachakova-Yordanova D, Baymakova M, Georgiev M, Mihova K, Petkova V, et al. Rare host variants in ciliary expressed genes contribute to COVID-19 severity in Bulgarian patients. Scientific Reports.14(1):19487 https://doi.org/10.1038/s41598-024-70514-3(2024).

3. Mir R, Ullah MF, Elfaki I, Alanazi MA, Algehainy NA, Altemani FH, et al. Whole-exome sequencing reveals rare genetic variants in saudi covid-19 patients with extreme phenotypes. Viruses.17(9):1198 https://doi.org/10.3390/v17091198(2025).

4. Khan A, Nayeem A, White N, Siddiqui A, Hussaini A, 22 CTRSKCUI, et al. Critical care trends across five waves of COVID-19 in Pakistan: A multi-center observational study from the Pakistan Registry of Intensive CarE (PRICE).26(1):803 2026).

Attachment Submitted filename: Response to reviewer.docx

# Peer review: Decision Letter 1

**DOI:** 10.1371/journal.pone.0360429.r003

**Authors:** Flora De Conto

27 Sep 2026

Whole-exome sequencing identifies a missense variant in the cytoskeletal gene SVIL implicating actin remodeling in COVID-19 severity in a Pakistani cohort

PONE-D-26-24088R1

Dear Dr. Khatoon,

We’re pleased to inform you that your manuscript has been judged scientifically suitable for publication and will be formally accepted for publication once it meets all outstanding technical requirements.

Within one week, you’ll receive an e-mail detailing the required amendments. When these have been addressed, you’ll receive a formal acceptance letter and your manuscript will be scheduled for publication.

An invoice will be generated when your article is formally accepted. Please note, if your institution has a publishing partnership with PLOS and your article meets the relevant criteria, all or part of your publication costs will be covered. Please make sure your user information is up-to-date by logging into Editorial Manager at Editorial Manager® and clicking the ‘Update My Information' link at the top of the page. For questions related to billing, please contact billing support.

If your institution or institutions have a press office, please notify them about your upcoming paper to help maximize its impact. If they’ll be preparing press materials, please inform our press team as soon as possible -- no later than 48 hours after receiving the formal acceptance. Your manuscript will remain under strict press embargo until 2 pm Eastern Time on the date of publication. For more information, please contact onepress@plos.org.

Kind regards,

Flora De Conto, Ph.D.

Academic Editor

PLOS One

Reviewers' comments:

Reviewer's Responses to Questions

**Comments to the Author**

Reviewer #1: All comments have been addressed

**********

2. Is the manuscript technically sound, and do the data support the conclusions?

Reviewer #1: Yes

**********

3. Has the statistical analysis been performed appropriately and rigorously?

Reviewer #1: Yes

**********

4. Have the authors made all data underlying the findings in their manuscript fully available?

The PLOS Data policy

Reviewer #1: Yes

**********

5. Is the manuscript presented in an intelligible fashion and written in standard English?

Reviewer #1: Yes

**********

Reviewer #1: I am satisfied with all the changes rendered.The manuscript has had those quesions honored.

The figures may be of high resolution though

**********

what does this mean?). If published, this will include your full peer review and any attached files.

If you choose “no”, your identity will remain anonymous but your review may still be made public.

**Do you want your identity to be public for this peer review?**  For information about this choice, including consent withdrawal, please see our Privacy Policy

Reviewer #1: **Yes:**  Prashanth N Suravajhala

**********

# Editor report: Acceptance letter

**DOI:** 10.1371/journal.pone.0360429.r004

**Authors:** Flora De Conto

PONE-D-26-24088R1

PLOS One

Dear Dr. Khatoon,

I'm pleased to inform you that your manuscript has been deemed suitable for publication in PLOS One. Congratulations! Your manuscript is now being handed over to our production team.

At this stage, our production department will prepare your paper for publication. This includes ensuring the following:

* All references, tables, and figures are properly cited

* All relevant supporting information is included in the manuscript submission,

* There are no issues that prevent the paper from being properly typeset

You will receive further instructions from the production team, including instructions on how to review your proof when it is ready. Please keep in mind that we are working through a large volume of accepted articles, so please give us a few days to review your paper and let you know the next and final steps.

Lastly, if your institution or institutions have a press office, please let them know about your upcoming paper now to help maximize its impact. If they'll be preparing press materials, please inform our press team within the next 48 hours. Your manuscript will remain under strict press embargo until 2 pm Eastern Time on the date of publication. For more information, please contact onepress@plos.org.

You will receive an invoice from PLOS for your publication fee after your manuscript has reached the completed accept phase. If you receive an email requesting payment before acceptance or for any other service, this may be a phishing scheme. Learn how to identify phishing emails and protect your accounts at https://explore.plos.org/phishing.

If we can help with anything else, please email us at customercare@plos.org.

Thank you for submitting your work to PLOS One and supporting open access.

Kind regards,

PLOS One Editorial Office Staff

on behalf of

Prof. Flora De Conto

Academic Editor

PLOS One
